# A Physics-Inspired Approach to Improve Oligonucleotide Design and Gene Synthesis

**DOI:** 10.34133/csbj.0189

**Published:** 2026-08-06

**Authors:** David Luna-Cerralbo, Ana Serrano, Fadi Hamdan, Juan Martínez-Oliván, Pierpaolo Bruscolini

**Affiliations:** ^1^ Departamento de Física Teórica, Facultad de Ciencias, Universidad de Zaragoza, Zaragoza 50009, Spain.; ^2^ Instituto de Biocomputación y Física de Sistemas Complejos (BIFI), Universidad de Zaragoza, Zaragoza 50018, Spain.; ^3^ Certest Pharma, Certest Biotec S.L, Polígono Industrial Río Gállego II, Calle J, 1, San Mateo de Gállego 50840, Spain.

## Abstract

Polymerase cycling assembly (PCA) allows gene synthesis from the assembly of overlapping oligonucleotides. For optimal yield, the sets of overlapping fragments should present uniform hybridization temperatures and minimal spurious dimer formation, conditions that become particularly difficult to fulfill for short, GC-biased, or dimer-prone sequences. We present a new algorithm for designing overlapping oligonucleotides for PCA. Unlike heuristic-based methods, the approach maps the design onto a statistical physics model whose low-temperature solution can be obtained exactly, through a transfer-matrix formalism, when spurious heterodimer interactions are negligible; when they are not, it is complemented by a simulated-annealing repair step and, optionally, by codon redesign, generating high-quality, thermodynamically consistent oligonucleotide sets. We evaluated the method on over 10,000 sequences previously deemed unsuitable for synthesis, successfully recovering roughly 80% of them, and more than 91% if codon redesign is allowed. Experimental validation on a subset of 14 representative designs confirmed the absence of dimer formation, with all assemblies yielding clean bands in agarose gel electrophoresis. Our approach offers a robust and reliable solution for oligonucleotide design, particularly in challenging sequence contexts, and represents a valuable tool for synthetic biology and automated gene assembly workflows.

## Highlights

• A new design method is proposed, which maps the search for the optimal oligo design into the study of the equilibrium state of a statistical physics model. If heterodimers are negligible, our exact solution represents the globally optimal design, given the specified experimental parameters and oligonucleotide lengths.
• When heterodimers cannot be neglected, we show that simulated annealing simulations are able to find a dimer-free solution in most of the cases; codon redesign represents a further tool, when the simulated annealing fails, to rescue the sequences that are still affected by heterodimers.
• The 3 approaches are integrated into a web server, CertPhys, capable of providing a reliable design for gene synthesis even in hard cases.

## Graphical Abstract

**Figure:**
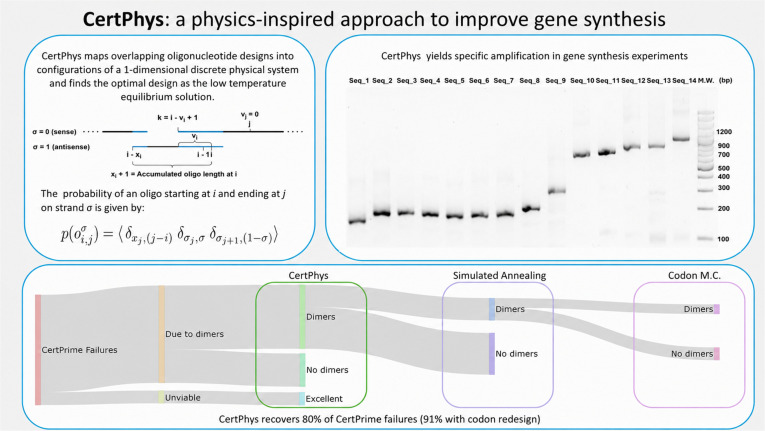

## Introduction

The ability to synthesize genes has become a cornerstone of molecular biology, providing researchers with the means to construct long, customized DNA sequences with high precision. This capability has revolutionized various fields, particularly in medicine, where it plays a crucial role in gene therapies [1], the production of recombinant proteins for targeted treatments [2,3], and the development of mRNA vaccines [4]. The latter, in particular, has had a transformative impact on global health, as demonstrated by its role in combating the COVID-19 pandemic. The predominant approach to gene synthesis relies on polymerase chain reaction (PCR) using overlapping oligonucleotides. This method, called polymerase cycling assembly (PCA) or Assembly PCR, involves designing short DNA fragments with complementary regions that anneal to one another, allowing DNA polymerase to extend and assemble them into a full-length sequence. To ensure an efficient and uniform assembly process, the melting temperatures (*T*_m_) of these overlapping regions must be closely matched and aligned with an optimal target value ($T_{m}^{*}$), which depends on the working temperature of the DNA polymerase [5]. Although matching *T*_m_ values does not, by itself, guarantee seamless amplification, it is a fundamental requirement in all computational design strategies. Consequently, every oligonucleotide design tool integrates melting temperature calculations, although the specific methodologies and constraints vary among them. Such melting temperatures do not depend only on the sequence of the overlapping regions, but also on other experimental parameters, including DNA concentration, ionic strength, and dNTP (deoxyribonucleotide triphosphate) levels, that substantially impact PCR efficiency and oligonucleotide hybridization [6]. While some tools offer limited customization of these parameters, most lack comprehensive control over factors such as monovalent and divalent ion concentrations, which can lead to deviations in melting temperatures and, ultimately, inefficient amplification.

Computational design strategies for oligonucleotide assembly generally follow 2 approaches: gapless PCR and gapped PCR. In gapless PCR, the oligonucleotides on each strand are contiguous, ensuring high fidelity during synthesis. Gapped PCR, on the other hand, introduces intentional gaps between oligonucleotides on the same strand, relying on complementary overlaps from the opposite strand to fill in missing segments. This alternative approach offers increased flexibility and cost-effectiveness for specific applications. Several bioinformatics tools have been developed to support these strategies. For gapless PCR, widely used programs include TmPrime [7] and DNAWorks [8]. For gapped PCR, tools such as Gene2Oligo [9], GeneDesign [10], Assembly PCR OligoMaker [11], Primerize [12], Integrated [13], and DeepFirstSearch [14] are commonly used. Whatever the gapped or gapless approach, most tools face several limitations, including difficulties in handling long DNA sequences, inadequate control over oligonucleotide length, and challenges in mitigating unwanted dimer formation. All these issues strongly affect the design quality, sometimes obliging a change in the experimental protocol. This is the case, for instance, of the challenge represented by long DNA sequences. Some tools, such as Primerize and Integrated, either lack support for long sequences or experience exponential increases in computational time as sequence length grows, making them impractical for large-scale projects.

Another fundamental constraint in oligonucleotide design is length optimization. Chemically synthesizing long oligonucleotides is challenging due to increased synthesis errors and lower yields [15]. Some tools, such as Primerize and PCR OligoMaker, allow users to specify length constraints, but others, like DeepFirstSearch and Integrated, do not provide this flexibility, often defaulting to shorter oligonucleotides. While shorter oligos reduce synthesis-related errors, they require a larger number of fragments to assemble the same DNA sequence, increasing complexity in laboratory workflows.

The formation of unintended oligonucleotide dimers is another major hurdle in gene synthesis. Spurious interactions, such as homodimers (self-pairing within an oligonucleotide) and heterodimers (unintended pairing between different oligonucleotides), can lead to nonspecific amplification and reduced efficiency. Some design tools, such as Integrated and DeepFirstSearch, incorporate algorithms to minimize dimer formation. In contrast, PCR OligoMaker does not include such functionality, while Primerize merely provides warnings about potential dimerization, requiring manual intervention from the user to optimize the design.

To overcome the above limitations, we have recently introduced CertPrime [16], an oligonucleotide design tool specifically developed to enhance computational efficiency and experimental precision in gene synthesis. CertPrime efficiently handles long DNA sequences, provides extensive customization options for experimental parameters (including ion concentrations, DNA concentration, and dNTP levels), and allows users to define maximum oligonucleotide lengths to align with synthesis constraints. In this way, CertPrime directly addresses the 3 main limitations of earlier tools highlighted above: It processes long sequences efficiently, lets the user bound the oligonucleotide length, and reduces spurious dimers through a post-design filtering that rejects designs with homodimer- or heterodimer-prone oligonucleotides. In comparative analyses, CertPrime outperformed tools such as PCR OligoMaker and Primerize, achieving a balance between accurate hybridization temperature predictions and an optimal number of oligonucleotides. However, CertPrime is based on an heuristic algorithm, which is not guaranteed to find the best design, but only a locally optimal one (i.e., the solution is a local minimum of a cost function, and not the global best solution). While this is not a problem in most of the cases, since CertPrime’s designs are good enough to implement a very efficient amplification and synthesis, there are some genes that CertPrime is not able to “resolve” into an oligonucleotide design with the desired quality.

To improve on this aspect, here we address the design problem with a different approach, mapping it to a statistical physics problem, so that the optimal design is mapped to the thermodynamic equilibrium of a one-dimensional discrete-variables system, whose energy encodes the cost function of the design. Limiting the range of interactions to near neighbors forfeits the possibility to address heterodimers, but allows the system to be solved exactly, through a transfer-matrix formalism. Thus, the predicted low-temperature solution will correspond to the globally optimal design (yet, disregarding heterodimers, that should be checked separately). Such solution is unique, unless some symmetry in the sequence makes it possible for 2 different design to have the same energy, which is quite unlikely in real cases, as we will see in the following.

We tested this method on the sequences where CertPrime failed, obtaining a notable improvement on CertPrime’s results, with comparable computational costs. While this choice might appear self-referential at first, it is justified on the grounds of 2 observations: First, CertPrime was previously tested against several other tools on a small set of sequences, and it was seen to outperform them. So, we estimate that the “hard” sequences for CertPrime might constitute a set of truly difficult sequences. Second, a thorough head-to-head comparison with those tools is not possible (several of them are no longer available as a web server or independent code), while a comparison with CertPrime could be conducted rigorously and efficiently.

In some cases, the obtained design is still problematic, because of the formation of heterodimers with melting temperatures close to the desired working temperature. In those cases, we apply a simulated annealing (SA) Monte Carlo (MC) approach, to see if an alternative solution exists, that trades some design quality for the absence of dimers. If the challenge is to perform an oligo design for a certain protein sequence, and not for a precise nucleotide sequence, codon redesign is also a possibility, complementary or alternative to the SA step. We test it for the sequences that are still dimer-prone after the SA protocol, and we are able to rescue roughly half of them. While these MC-based simulated-annealing and codon-redesign approaches increase the computational design-time, in practice this does not represent a serious problem, since this time is still a small fraction of the time required by wet-lab amplification and synthesis protocols.

For this reason, we regard this approach, that in the following we call “CertPhys” for short, as an important advancement toward more reliable oligonucleotide design for gene synthesis, particularly in the hard cases where heuristic tools struggle.

## Methods

### Model

**Fig. 1.**
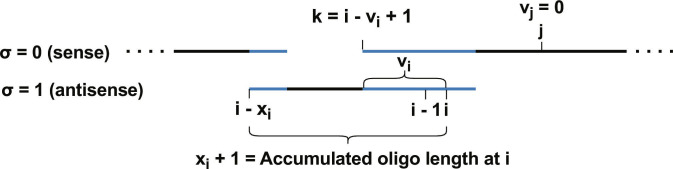
Model definition. The segments represent the oligonucleotides, with the blue parts corresponding to the overlapping regions. See text for details.

We map any possible oligonucleotide design in a unique configuration of the statistical physics model defined in the following way. Let *L* be the length of the DNA to be synthesized; we introduce a one-dimensional lattice of *L* sites, oriented in the $5^{\prime }\to 3^{\prime }$ direction of the sense strand (as in Fig. 1). The numbering of the oligonucleotides in the design will follow the natural order associated to this orientation of the lattice so that we will refer to the first and last oligo as the one that starts at *i* = 1 and the one that ends at i=L, respectively. To each site *i* of the lattice, we associate a collection of 3 discrete variables $\xi_{i}=\left(x_{i},v_{i},\sigma_{i}\right)$, with values in the ranges $x_{i}\in \left[0,l^{M}-1\right]$, $v_{i}\in \left[0,O^{M}\right]$, and $\sigma_{i}=0,1$, to be interpreted as follows: Position *i* falls inside an oligo on strand $\sigma_{i}$ = {0 (sense), 1 (antisense)} that started at position $\left(i-x_{i}\right)$; at the left of (and including) position *i* (i.e., on its 5′), such oligo accumulates an overlap of $v_{i}$ nucleotides with the following oligo in the design (on the other strand): i.e., the following oligo starts at position $k=i-v_{i}+1$ of the opposite strand. In the above, *l*^M^ indicates the maximum length allowed for any oligonucleotide in the design, and *O^M^* is the maximum length allowed for the overlap region between consecutive oligos. In the following, we will strictly refer to $o_{i,j}^{\sigma }$ as the nucleotide sequence starting at *i* and ending at *j* on strand *σ*; that is, if $o_{i,i+5}^{0}$ = ACCGTT in the sense strand, the sequence in the antisense strand is $o_{i,i+5}^{1}$ = TGGCAA (hence, it is written in the 3′→5′ direction).

The above encoding implies that the states at neighboring sites are not independent, but are restricted by a set of on-site and site-pair constraints, which we will call “realizability” constraints (detailed in Section S1.1), since they select only those states of the model that correspond to any and each possible design.

On such physical model, we can define an energy function that encodes the design problem so that the optimal design corresponds to the lowest energy state. We write the energy as the sum of a local ($H^{\prime }$) and a nonlocal term (*V*):

$$H=H^{\prime }+V=H_{0}+H_{1}+V$$ (1)

where:

• $H_{0}$ accounts for the energy of overlapping adjacent oligos on different strands (i.e., the “correct” interactions), providing a quadratic penalty if their melting temperature *T*_m_ departs from the target T^*^. It can be written as an interaction between neighboring sites as:
  $$\begin{array}{c} \begin{array}{l} H_{0}=\sum_{k=1}^{L-1}H_{0,k}\left(\xi_{k},\xi_{k+1}\right)\\ \;=\sum_{k=1}^{L-1}\epsilon_{k-v_{k}+1,k}\;\delta_{\sigma_{k+1},1-\sigma_{k}}{\left(T_{m}\left(\left[k-v_{k}+1,k\right]\right)-T^{*}\right)}^{2} \end{array} \end{array}$$ (2)
  where $T_{m}\left(\left[i,j\right]\right)$ is the melting temperature of the region between positions *i* and *j* (included), $\epsilon_{i,k}>0$ for all *i*, *k* is a position-dependent energy cost, and $\delta_{\sigma_{k+1},1-\sigma_{k}}$ indicates the Kronecker delta, here used to ensure that *k* represents the ending position of an oligo of the design;

• $H_{1}$ penalizes neighboring oligos that may present multiple competing overlaps between them (as in case or repeated sequence motifs, e.g., “ACACACAC”), as well as self-hybridization: $H_{1}=\sum_{k=1}^{L}H_{1,k}\left(\xi_{k},\xi_{k+1}\right)$, with:
  $$\begin{array}{c} \begin{array}{l} H_{1,k}\left(\xi_{k},\xi_{k+1}\right)=\left(\left(1-\delta_{k,L}\right)\delta_{\sigma_{k+1},1-\sigma_{k}}+\delta_{k,L}\right)\\ \times \left(\mu \;\theta \left(T_{m}\left(o_{k-x_{k},k}^{\sigma_{k}}\right)-T^{\mathrm{thr}}\right)+\mu^{\prime }1_{\left\{\xi_{k}\in F_{k}\right\}}\right) \end{array} \end{array}$$ (3)
  Here, $\mu ,\mu^{\prime }>0$ are energy penalties, $\theta \left(x\right)$ is the Heaviside step function ($\theta \left(x\right)=1$ if x>0 and 0 otherwise), and $T_{m}\left(o_{k-x_{k},k}^{\sigma_{k}}\right)$ indicates the melting temperature of the self-interacting oligo starting at $k-x_{k}$ and ending at *k* on strand $\sigma_{k}$; such a temperature must be calculated as the melting temperature of the most stable coupling of the oligo with itself. $T^{\mathrm{thr}}<T^{*}$ is a threshold temperature that we impose on spurious interactions so that they do not compete with the correct match between adjacent oligos. $1_{A}$ is the indicator function, that is, 1 if condition *A* is satisfied and 0 otherwise; here, we use it to penalize that the oligo ending at *k* belongs to a precalculated list of “prohibited” oligos $F_{k}$, on the basis of local periodicities, or other kind of unwanted characteristics for the oligonucleotides in the design.
• *V* is a nonlocal term, which accounts for the possible interaction of distant oligos, in the same or opposite strands, and penalizes the cases when such interactions are competing with the correct coupling (i.e., their $T_{m}$ coming too close to the target $T^{*}$). This can be written as:
  $$\begin{array}{c} \begin{array}{l} V=\mu \sum_{i}\sum_{j>i}\delta_{\sigma_{i+1},1-\sigma_{i}}\delta_{\sigma_{j+1},1-\sigma_{j}}\theta \left(j-x_{j}-i\right)\\ \;\theta \left(T_{m}\left(o_{i-x_{i},i}^{\sigma_{i}},o_{j-x_{j},j}^{\sigma_{j}}\right)-T^{\mathrm{thr}}\right) \end{array} \end{array}$$ (4)
  where the Kronecker deltas ensure that 2 oligos of the design end at *i* and *j*, respectively, and the first step function, enforcing that $j-x_{j}>i$, ensures that such oligos do not overlap. The last step function provides an energy penalty only when the highest melting temperature $T_{m}\left(o_{i-x_{i},i}^{\sigma_{i}},o_{j-x_{j},j}^{\sigma_{j}}\right)$ of all possible arrangements of the oligos spanning the region $i-x_{i},i$ on strand $\sigma_{i}$ and $j-x_{j},j$ on strand $\sigma_{j}$ exceeds the threshold temperature $T^{\mathrm{thr}}$; by “arrangement”, here we mean any possible alignment of the 2 oligos, also in the reverse direction, in order to exclude also “unnatural” interactions between parallel couplings of the strands.

The energy functions defined above depend on several parameters, that in our tests were chosen as follows: The melting temperatures were calculated as described in [16], using Primer3 as the fundamental tool; $\mu^{\prime }=0$ (we did not introduce any list of prohibited oligos); *μ* = 50. The parameters $\epsilon_{i,k}$ where chosen to depend on the local CG content, since it causes strong heterogeneities on the dependence of the melting temperature of a region on its length.

### The equilibrium solution of the model as the optimal design

Within the above framework, the oligo design problem is converted in finding the model state $\xi =\left\{\xi_{i},i=1,\ldots ,L\right\}$ that satisfies the realizability constraints, and minimizes the energy function *H* of Eq. (1). This global optimization task can be addressed by stochastic numerical approaches like SA, genetic algorithm, and others, with computational costs that will depend on the algorithm of choice and on the length and “hardness” of the gene to be designed. Indeed, SA will be our bottom-line method for the hardest cases; yet, as a heuristic method, SA is not guaranteed to find the global optimum.

For this reason, we start with a different approach, analogous to the one implemented in [17] for a different problem, and look for an exact solution of a simpler energy function.

#### Exact optimization (neglecting heterodimers)

We notice that the *V* term in Eq. (1) is the only one providing nonlocal interactions along the lattice; if we neglect it, and just check that spurious interactions do not affect the quality of the resulting design, we can apply some technique like dynamic programming that exactly yields the best design for the reduced energy function $H^{\prime }=H_{0}+H_{1}$, with a predictable computational cost, at the price of preventing homodimers, but not heterodimers, to appear in the best design. We observe also that such solution corresponds to the zero-temperature equilibrium of the model, whose general solution at nonzero temperatures will be given by the Boltzmann probability distribution

$$p\left(\xi ;\tau \right)=\frac{1}{Z}e^{-\beta H^{\prime }\left(\xi \right)}\;$$ (5)

if ξ satisfies the realizability constraints, and $p\left(\xi ;\tau \right)=0$ otherwise. Here, β=1/τ is the inverse “temperature”, and

$$Z=\sum_{\xi }1_{C\left(\xi \right)}e^{-\beta H^{\prime }\left(\xi \right)}$$ (6)

is the partition function, normalizing the Boltzmann weight so that the above expression is indeed a probability, satisfying $\sum_{\xi }p\left(\xi ;\tau \right)=1$. The term $1_{C\left(\xi \right)}$ guarantees that only states that satisfy the realizability constraints C contribute to the sum; notice also that τ is not the true temperature (that used in the design process), but a parameter that tunes how many different designs contribute with nonzero probability: For τ→0, the distribution gets peaked, and just the state that minimizes $H^{\prime }$ is populated.

Although this alternative approach appears less straightforward, it is still exactly solvable, through transfer matrix techniques. Intuitively, the transfer-matrix method sweeps along the sequence one position at a time and, through successive matrix multiplications, propagates the best partial design compatible with each local state; because the interactions in $H^{\prime }$ are local, this single sweep is guaranteed to recover the global optimum of the reduced model, at a computational cost that grows essentially linearly with the sequence length. Thus, the possibility to work at τ>0 makes it possible to evaluate the robustness of the design against alternative ones; moreover, it provides a reference distribution to use in SA or other heuristic optimization protocols, which can explicitly account for the nonlocal interaction *V* should the latter prove indispensable for a good design. Thus, in the following, we adopt such thermodynamic approach and calculate the optimal design from the zero-temperature limit of the expectation value of suitably designed observables. Namely, we consider the probability of finding an oligo starting at *i* and ending at *j* on strand σ:

$$p\left(o_{i,j}^{\sigma }\right)=\langle \;\delta_{x_{j},\;j-i}\;\delta_{\sigma_{j},\;\sigma }\;\delta_{\sigma_{j+1},\;1-\sigma }\;\rangle ,$$ (7)

where the averages $\langle •\rangle$ are calculated with the probability Eq. (5) above, which ignores the nonlocal interactions corresponding to heterodimers. Equation (7) represents the marginal probability, i.e., the sum of the probabilities of all possible designs that contain oligo $o_{i,j}^{\sigma }$. However, if the best solution is unique (as we expect, in the absence of any symmetry), at *τ* = 0 there is only one configuration $\xi =\left\{\left(x_{j},v_{j},\sigma_{j}\right)\right\}$with nonzero probability (actually, with probability 1). So, we can single out the optimal oligo design, studying the β→∞ limit of the probabilities above, and simply selecting the values of *i*, *j*, and σ such that the above marginals are close to one.

In the unlikely case that the optimal solution is not unique, a MC simulation using these marginals as a background probability should be very efficient in finding all good solutions. However, in practice, we have not found such cases in our analysis.

#### Dealing with heterodimers: SA

If the design obtained through Eq. (5) does not contain any oligo pair with hybridization temperature $T_{m}>T^{\mathrm{thr}}$, then *V* = 0 on that configuration: Such design is the global minimum also of the general energy *H* of Eq. (1), and hence, it is the overall best design, given the input parameters. However, if heterodimer-prone oligonucleotides appear in the design obtained with $H^{\prime }$, then V>0 on it, and we cannot be sure that such design is a good solution (not even a local optimum). Hence, it must be discarded and the nonlocal term *V* must be accounted for in the minimization of the energy function Eq. (1). In that case, the transfer matrix approach is no longer viable, and we resort to SA simulations to look for the optimal solution.

In this framework, an alternative description of the model configurations is more handy, to avoid dealing with complex update rules.

**Fig. 2.**
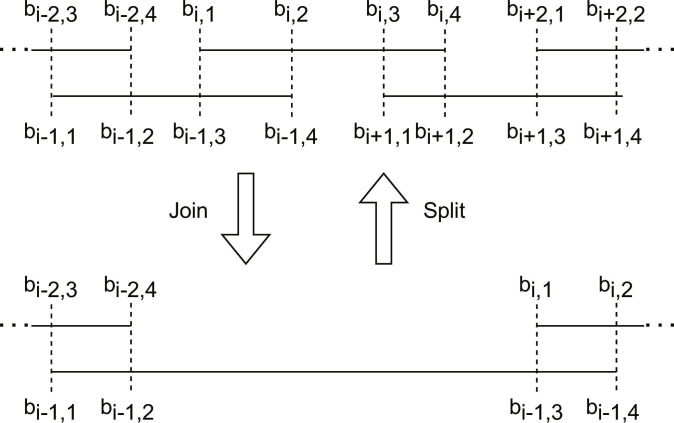
Schematic representation of the relative positions involved in the merge and split oligo movements.

Instead of the site-variables $\xi_{j}=\left(x_{j},v_{j},\sigma_{j}\right)$ , with j=1,…,L used previously, for each oligonucleotide *i* we introduce $\eta_{i}=\left(b_{i,1},b_{i,2},b_{i,3},b_{i,4}\right)$ such that $b_{i,1}\leq b_{i,2}<b_{i,3}\leq b_{i,4}$, representing the boundaries that define the upstream overlap with oligo (*i* − 1), $O_{i}^{↑}=\left[b_{i,1},b_{i,2}\right]$, the core region $C_{i}=\left[b_{i,2}+1,b_{i,3}-1\right]$ (not involved in overlaps), and the downstream overlap region with the following oligo $O_{i}^{↓}=\left[b_{i,3},b_{i,4}\right]$. For consistency (see Fig. 2, top), the following relations hold: $b_{i,1}=b_{i-1,3}$, $b_{i,2}=b_{i-1,4}$, $b_{i,3}=b_{i+1,1}$, and $b_{i,4}=b_{i+1,2}$.

The initial and final oligos have further restrictions: $b_{1,1}=b_{1,2}=0$, $b_{n,3}=b_{n,4}=L+1$; also, the number of oligos, *n*, is even, to guarantee that the first and last oligos lie on opposite strands. Thus, the set $\eta =\left\{\eta_{i},i=1,\ldots ,n\right\}$, with even *n*, describes completely the state of the system.

The MC simulation at each temperature is built on the following set of local (number-preserving) and nonlocal (number-changing) moves:

1. Overlap-boundary moves, which shift the position of 1 or 2 boundaries of an oligo by Δ nucleotides, either altering or preserving the overlap or core region’s length
2. Oligo addition/removal movements, which are carried out according to the scheme shown in Fig. 2.
  *Join* removes an intermediate core and its flanking overlaps, decreasing the count by 2; *Split* inserts a new core and 2 overlaps within the core of an existing oligo, increasing the oligo count by 2.

The combination of local slides/tweaks and split/join moves renders the Markov chain irreducible and aperiodic over the feasible state space, thus enabling exploration of any admissible segmentation. Notice that the above moves preserve the parity of the total number of oligos *n*, ensuring that *n* is even at every time.

The energy function used in the SA process is slightly different from that in Eq. (1). In the previous approach, constraints on minimum and maximum oligo lengths were considered at the partition function level, that is, as if their energy penalties were infinite. In a MC scheme, a better choice is to allow a finite cost for such violations, to increase the acceptance ratio of the movements, at least at high temperatures. We therefore write the energy as:

$$H_{\mathrm{MC}}=H+\Psi ,$$ (8)

where H is the base Hamiltonian from Eq. (1). The boundary term $\Psi =\Psi_{\text{oligo}}+\;\Psi_{\mathrm{ovl}},$with

$$\begin{array}{c} \Psi_{\text{oligo}}=\sum_{k=1}^{n}V^{\text{soft}}\left(L_{k},l^{m},l^{M},M_{L},\lambda_{L}\right), \end{array}$$ (9)

$$\begin{array}{c} \Psi_{\mathrm{ovl}}=\sum_{k=1}^{n-1}V^{\text{soft}}\left(|O_{k}^{↓}|,O^{m},O^{M},M_{O},\lambda_{O}\right), \end{array}$$ (10)

penalizes oligo and overlap lengths that fall out of the admitted ranges $\left[l^{m},l^{M}\right]$ and $\left[O^{m},O^{M}\right]$, respectively. Here, $L_{k}=\left(b_{k,4}-b_{k,1}\right)$ is the length of oligo k, $|O_{k}^{↓}|=\left(b_{k,4}-b_{k,3}\right)$ is the downstream overlap length for oligo *k*, and we have defined:

$$\begin{array}{c} V^{\text{soft}}\left(x,a,b,m,\lambda \right)=\left(m+\lambda \left(a-x\right)\right)1_{x<a}+\left(m+\lambda \left(x-b\right)\right)1_{x>b}. \end{array}$$ (11)

This expression ensures a fixed penalty m>0 plus a cost that is proportional to the extent of the constraint violation, whenever the lengths do not fall in the allowed range.

The SA protocol consists of a Metropolis–Hastings MC simulations performed at 20 decreasing temperature $\tau_{k}$, from $\tau_{0}=100$ to $\tau_{19}=0.01$, with $n_{S}=20,000$ MC steps at each temperature.

Each MC move $\eta \to \eta^{\prime }$ is accepted according to a Metropolis–Hastings criterion:

$$\begin{array}{c} \alpha \left(\eta \to \eta^{\prime }\right)=\min\;\left\{1,\frac{q\left(\eta^{\prime }\to \eta \right)}{q\left(\eta \to \eta^{\prime }\right)}e^{-\frac{1}{\tau }\left(H_{\mathrm{MC}}\left(\eta^{\prime }\right)-H_{\mathrm{MC}}\left(\eta \right)\right)}\right\} \end{array}$$ (12)

In this framework, we can take advantage of the knowledge of the exact solution for the simplified model, using Eq. (7) as a background probability associated to the elementary move proposal $q\left(\eta \to \eta^{\prime }\right)$.

Futher details of the SA protocol can be found in Section S1.2.

### Sequence redesign

When both the standard design procedure and the SA repair layer fail to yield a feasible construct—typically in highly constrained regions where the original coding sequence is prone to strong dimerization, extreme GC bias, or extended self-complementarity—the only remaining option is to address a softer version of the problem: allowing controlled modifications of the nucleotide sequence while preserving the encoded protein. In such cases, we employ a dedicated MC algorithm that performs synonymous codon substitutions within the reading frame, thereby maintaining the amino acid sequence while exploring alternative codon configurations with improved physicochemical properties.

Since human coding sequences serve as our test benchmark, this strategy was implemented using the MC codon-optimization framework introduced in [18]. This model generates human-like coding sequences that accurately reproduce both single-codon usage frequencies and codon-pair statistics characteristic of the human transcriptome (although, in practice, the codon-usage pattern can be adapted to any target species). All simulations were performed at an inverse temperature of β=1, corresponding to the natural-temperature regime of the model, which provides a realistic balance between stochastic exploration and adherence to empirical codon preferences.

In order to tailor the codon-redesign process to the requirements of oligo design—and specifically to eliminate sequence features known to jeopardize gene synthesis—we incorporated an additional filtering stage during sampling. Each time a candidate sequence is accepted as a MC move, it is immediately screened for the following undesirable patterns:

1. Local GC imbalance: GC content computed over sliding windows of length $L_{\mathrm{GC}}$ [typically 50 nucleotides (nt)] must lie within $\left[{\mathrm{CG}}_{\min},{\mathrm{CG}}_{\max}\right]$.
2. Direct repeats: Stretches of identical nucleotides longer than $L_{r}$ (typically *7* nt). Such homopolymeric runs can cause polymerase slippage and destabilize oligo binding.
3. Complementary runs: Contiguous regions longer than $L_{c}$ (typically 20 nt) where the sequence is complementary to another region of the same strand, promoting intramolecular hairpin formation.
4. Reverse-complementary runs: Contiguous regions longer than $L_{\mathrm{rc}}$ (typically 20 nt) that are reverse-complementary to another segment within the sequence, increasing the likelihood of stable secondary structures or unwanted self-dimerization.
5. Restriction sites: Subsequences corresponding to restriction enzyme recognition motifs (or their reverse complements), which were already excluded in [18].

The protocol follows a first-feasible (first-passage) stopping rule: As soon as a newly generated codon sequence satisfies all the filtering criteria above, the Monte Carlo process is terminated and the accepted sequence proceeds to the oligo-design stage, using the transfer-matrix and simulated-annealing procedures described earlier.

### Short-primers design

In order to amplify the whole gene and prevent partial or incomplete amplification, it is important that the experimental PCR protocol may rely on 2 primers that anneal to the 3′ ends of the template and complementary strands, interacting with the first and last oligonucleotides of the design with the highest probability. Here, we use the same strategy adopted in CertPrime to design “short” oligo primers, fully complementary to the first and last oligo of the optimal design found by CertPhys, with a melting temperatures of the oligo as a whole ($T_{m}$(S.O.)) close to a target value ($T^{*}$(S.O.)), and a given minimum length ($l^{m}$ (S.O.)).

Namely, we take the first (last) *k* bases of the initial (final) oligo, with *k* equal to the minimum oligo length for short oligonucleotides ($l^{m}$ (S.O.)). Then, we continue adding bases until the distance between the melting temperature of the short oligo ($T_{m}$(S.O.)) and the target value is minimized (yet avoiding overlaps with the second or penultimate oligonucleotide of the design).

### Input parameters

The complete set of input parameters that, together with the gene sequence, are needed by CertPhys is reported in Table 1, along with the values employed in all computational tests presented in this work. Table 2 lists the additional input parameters that need to be specified if the codon redesign protocol is applied.

**Table 1.** Input parameters. User-defined parameters required by CertPhys. The reported values correspond to those used in the computational tests. $T^{*}$ is the target melting temperature for overlap regions; [*l^m^, l^M^*] denotes the allowable length range for any oligonucleotide in the design; [*0^m^, 0^M^*] specifies the range of possible overlap lengths. $T^{\mathrm{thr}}$ is the threshold temperature above which spurious dimer interactions are considered a threat for the design. Ionic concentrations are indicated in brackets. [Na^+^], [Mg^2+^], [DNA], and [dNTPs] are required by Primer3 to compute the melting temperature of each oligo sequence (see the Model section). $T^{*}$(S.O.) and l^m^(S.O.) refer to the target temperature and minimal length for the short oligonucleotides (see the Short-primers design section).

**[Na^+^]**	50 mM	$T^{*}$	60 °C
**[Mg^2+^]**	1.5 mM	*T*^thr^	55 °C
**[dNTPs]**	0.6 mM	[*O^m^, O^M^*]	15–30 bp
**[DNA]**	300 nM	[*l^m^, l^M^*]	45–65 bp
$T^{*}$ **(S.O.)**	60 °C	*l^m^* (S.O.)	16 bp

**Table 2.** Input parameters for codon redesign. Default thresholds used by the codon-level Monte Carlo procedure to penalize undesirable sequence features. *L_r_* is the maximum allowed length for exact direct repeats; *L_c_* and *L_rc_* are the maximum lengths for complementary and reverse-complementary runs, respectively. Local GC content is evaluated in sliding windows of length *L_CG_* and must lie within the admissible range [*CG_min_*, *CG_max_*].

***L_r_***	7 bp	*L_CG_*	50 bp
***L_c_***	20 bp	*CG_max_*	70%
***L_rc_***	20 bp	*CG_min_*	30%

### Method-assessment protocol

#### Human genome database preparation

To perform an in silico evaluation of our method, we reused the dataset originally compiled for CertPrime [16]. These 73,424 sequences were downloaded from the *Homo sapiens* database hosted by Ensembl [19] on 2024 April 10 and ranged in length from 150 to 1,350 nt.

Two distinct databases were derived from this dataset, each tailored for a specific evaluation goal. The first one, used to assess the design capability of our algorithm, comprises 10,853 sequences from the CertPrime dataset for which that method failed to produce viable oligonucleotide designs. These sequences either exhibited spurious dimer formation or were classified by CertPrime as nonviable according to its internal quality metrics.

The second database was designed to evaluate the computational efficiency of our approach. For this purpose, we randomly sampled 1,000 sequences from CertPrime’s original 73,424 sequences database, selecting sequences with lengths uniformly distributed between 150 and 1,000 nt. This sampling strategy allowed us to benchmark the computational cost of our method across a broad and representative range of sequence lengths. This range was chosen because it covers the sizes most relevant to single-fragment oligonucleotide assembly: Sequences much shorter than 150 nt seldom require a multi-oligo design, whereas longer genes are, in standard laboratory practice, split into shorter blocks that are assembled separately and subsequently joined so that the per-block design problem typically remains within this window.

#### Quality metrics for oligonucleotide designs

To assess the quality of each oligonucleotide design, we used the same quality indicators and classification criteria used in CertPrime [16]. Specifically, we adopted the following metrics from that approach:

• Temperature range ($\Delta T_{m}$): the difference between the maximum and minimum melting temperatures observed within a design.

$$\Delta T_{m}=T_{m}^{\max}-T_{m}^{\min}$$ (13)

• Deviation (*ρ*) from the target temperature T^*^: It measures the overall deviation of melting temperatures from the user-specified target temperature:

$$\rho =\sqrt{\frac{1}{n-1}\sum_{i=1}^{n-1}{\left(T^{*}-T_{m}^{i}\right)}^{2}}$$ (14)

• Temperature spread (*σ*): The standard deviation of melting temperatures around their mean value, reflecting the uniformity of the melting temperature across the design:

$$\sigma =\sqrt{\frac{1}{n-1}\sum_{i=1}^{n-1}{\left({\bar{T}}_{m}-T_{m}^{i}\right)}^{2}}$$ (15)

Here, *n* represents the total number of oligonucleotides in a design, and $T_{m}^{i}$ denotes the melting temperature of the hybridization region between oligonucleotides *i* and i+1. ${\bar{T}}_{m}$ is the average melting temperature calculated as ${\left(n-1\right)}^{-1}\sum_{i=1}^{n-1}T_{m}^{i}$, while $T_{m}^{\min}$ and $T_{m}^{\max}$ correspond to the minimum and maximum melting temperatures found in the design, respectively.

Based on these metrics, CertPrime’s classification defined oligonucleotide designs as:

• Excellent: ρ<1.5 and σ<1 and $\Delta T_{m}<2$;
• Good: ρ<2.5 and σ<2 and $\Delta T_{m}<3$, but not qualifying as “Excellent”;
• Viable: ρ<3.5 and σ<3 and $\Delta T_{m}<4$, but not qualifying as “Good”.

For consistency, here we use the same classification.

#### Synthesis of DNA sequences

Oligonucleotides were synthesized using the phosphoramidite method [20] on an automated Mermade 192E synthesizer (LGC Biosearch Technologies) and purified by desalting.

For short sequences, assembly and amplification were performed in one step. PCR was performed in a final volume of 25 μl, with 40 pmol of each outer oligo and 1 pmol of each inner oligo, 100 mM KCl, 3% dimethyl sulfoxide (DMSO), and 1× PrimeSTAR Max Premix (Takara). The reaction mixture was subjected to the following thermal protocol: initial denaturation at 98 °C for 30 s, followed by 20 cycles of denaturation at 98 °C for 5 s, annealing at 60 °C for 10 s, and extension at 72 °C for 30 s. A final extension step at 72 °C for 5 min was performed after the last cycle.

For sequences longer than 500 base pairs (bp), a second PCR was performed to ensure amplification of the full-length sequence and minimized undesired subproducts. For this second PCR, 2 μl of the crude product from the first PCR was mixed with 40 pmol of short primers, 100 mM KCl, 3% DMSO, and 1× PrimeSTAR Max Premix (Takara) in a final volume of 25 μl. The thermal cycling conditions were as follows: initial denaturation at 98 °C for 30 s; 30 cycles of denaturation at 98 °C for 5 s, annealing at 60 °C for 10 s, and extension at 72 °C for 30 s; followed by a final extension at 72 °C for 5 min. PCR products were purified using QIAquick PCR Purification Kit and then analyzed using 2% agarose gel electrophoresis. Sequences were verified by Sanger sequencing.

## Results

### Recovery of CertPrime failures

For the 10,853 human coding sequences for which CertPrime failed to design oligonucleotides, 2 distinct kinds of failure were identified: (a) 1,269 sequences (11.7%) did not meet CertPrime’s minimum quality thresholds and were deemed nonviable according to the thermal metrics defined in the Quality metrics for oligonucleotide designs section, and (b) 9,584 sequences (88.3%) were rejected because their candidate oligos were predicted to form disruptive homo- or heterodimerization.

**Fig. 3.**
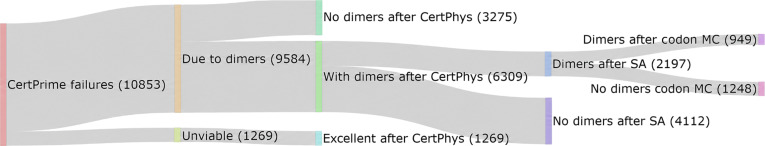
Workflow for sequence evaluation and optimization. Sankey diagram summarizing the multi-stage rescue of dimer-prone designs. Node widths are proportional to the number of sequences transitioning between stages; colors denote classification status after each step.

The CertPhys protocol allows to recover most of the sequences, as summarized in the Sankey diagram (Fig. 3). For designs rejected solely on the grounds of the thermal-quality criteria, we redesigned the sequences with CertPhys and quantified the proportion recovered (see the next section). For designs rejected due to the predicted risk of dimerization, we likewise attempted a redesign with CertPhys. Sequences that continued to exhibit problematic off-target dimerization were subsequently processed by the SA repair layer described in the Dealing with heterodimers: SA section. Finally, sequences that still failed after repair were considered intrinsically challenging; for these, we applied the sequence-redesign MC described in the Sequence redesign section, which perturbs codon usage while preserving the encoded protein.

#### Recovery of underperforming sequences

**Fig. 4.**
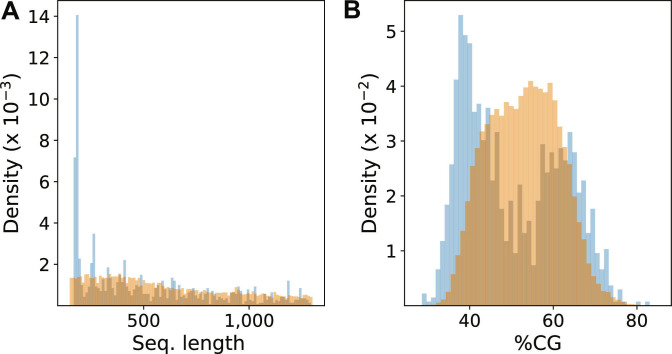
Comparison between sequences rejected by CertPrime and the full dataset. (A) Distribution of sequence lengths in the full dataset (orange) versus the subset of 1,269 sequences rejected by CertPrime (blue). Short sequences are clearly overrepresented among the failures. (B) GC-content distributions for the same 2 sets. The rejected sequences show a higher frequency of GC-extreme regions (below 40% or above 60%).

For the first group of 1,269 sequences, CertPrime’s designs did not meet the thermal quality thresholds required for viability. These designs exhibited excessive temperature dispersion, high deviation from the target melting temperature, or both (see the Quality metrics for oligonucleotide designs section). To understand the reasons of these failures, we analyzed the sequence composition and design profiles of this subset in comparison with the full set of 10,853 original targets. As shown in Fig. 4, 2 notable trends emerged: First, a sequence-length bias appears: design failures are much more common for short sequences, with less than 250 nt; second, a clear dependence on GC content: a large proportion of the rejected sequences had average GC fraction either below 40% or above 60%, indicating that CertPrime’s heuristics perform poorly when the nucleotide composition deviates substantially from a balanced (≈50%) profile.

These 2 properties—short sequence length and GC imbalance—introduce frustration in the configuration space, which challenges the local, heuristic algorithm of CertPrime. Indeed, short sequences imply more difficulties in satisfying the constraints on oligo lengths while coping with thermodynamic requirements; on the other hand, extreme CG contents affect the overlap lengths through the melting temperatures, again setting the optimization algorithm on the horns of a dilemma, between overlap lengths and thermodynamic requirements.

In such constrained scenarios, where the design space is narrow and tolerance margins are low, CertPrime’s heuristic-driven approach often fails to identify suitable configurations.

In contrast, provided that a heterodimer-free solution exists, CertPhys is able to find it, by applying the global optimization framework rooted in statistical physics. As a result, CertPhys successfully generated viable designs for all 1,269 sequences previously rejected due to thermal quality issues, achieving an Excellent classification in every case, as reflected in the Sankey diagram (Fig. 3). It is interesting to notice that the best solution by CertPhys usually presents a larger number of shorter oligos than that by CertPrime (Fig. S2). This finer granularity enabled tighter control over local melting temperatures, reducing both inter-oligo variability and global deviation from the target temperature, thereby satisfying quality thresholds even in compositionally challenging contexts.

**Fig. 5.**
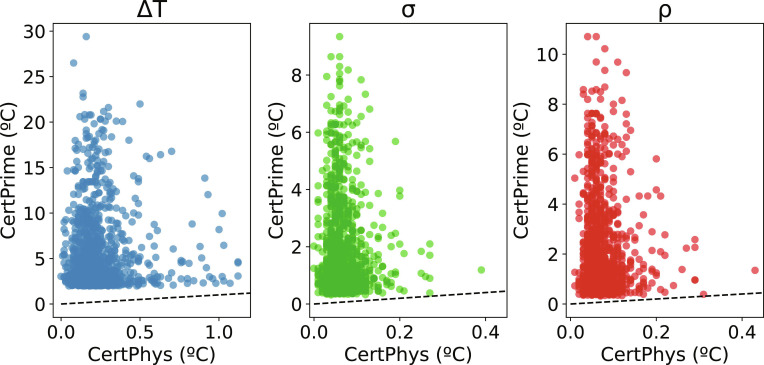
Pairwise comparison of thermal quality metrics between CertPrime (*y* axis) and CertPhys (*x* axis) for the 1,269 sequences rejected by CertPrime. Each point corresponds to one sequence. Lower values indicate higher thermal uniformity and thus improved design quality. A dashed line signals the y=x locus: Points above it represent sequences for which the design by CertPhys is better than that by CertPrime.

To quantify the recovery of the 1,269 sequences, we compared the key quality metrics—$\Delta T_{m}$, σ, and ρ—obtained by CertPrime and CertPhys for each case (Fig. 5). In particular, CertPhys reduced the temperature range $\Delta T_{m}$ from values as high as 30 °C to values below 1 °C, the temperature variability *σ* from over 8 °C to under 0.4 °C, and the deviation from target *T*_m_, *ρ*, from above 10 °C to less than 0.4 °C in nearly all cases.

#### Resolution of dimer-related failures

We next addressed the largest subset of failures: the 9,584 sequences rejected by CertPrime due to predicted dimerization at or above $T^{\mathrm{thr}}=55^{\circ }C$ . To evaluate the recovery capability of CertPhys, these sequences were redesigned under default parameters. This initial redesign successfully resolved dimer-related issues in 3,275 cases, generating high-quality oligonucleotide sets that met the Excellent classification criteria.

**Fig. 6.**
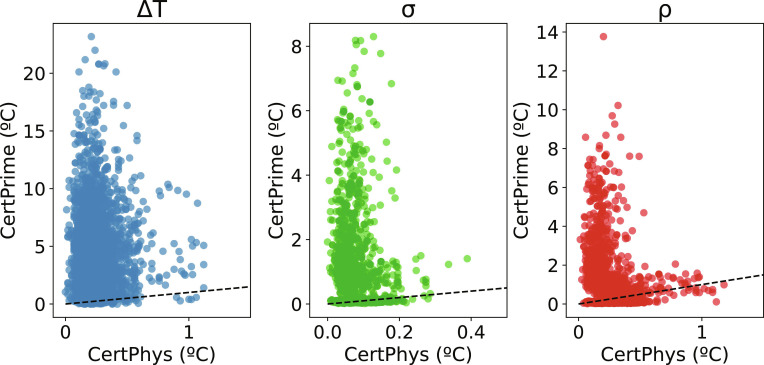
Pairwise comparison of thermal quality metrics between CertPrime (*y* axis) and CertPhys (*x* axis) for the 3,275 dimer-prone sequences recovered by CertPhys. Each point corresponds to one sequence. Lower values indicate higher thermal uniformity and thus improved design quality. A dashed line signals the y=x diagonal.

For such sequences, Fig. 6 compares the thermal quality metrics described in the Quality metrics for oligonucleotide designs section between the original CertPrime designs (with dimers) and the redesigned CertPhys solutions (without dimers). Despite the removal of dimerization constraints, CertPhys achieved improved values for $\Delta T_{m}$, *σ*, and *ρ* relative to CertPrime in most of the cases, as can be seen by noticing that the majority of points falls above the dashed line representing the y=x diagonal of the graph. This indicates that CertPrime tended to compromise thermal performance in an unsuccessful attempt to suppress dimer formation, whereas CertPhys avoided such conflicts from the outset by exploring configurations with a larger number of oligos per design. Conversely, the *ρ* values obtained for this subset were higher than those observed in Fig. 5, reflecting the inherently greater difficulty of designing thermally uniform oligos for highly dimer-prone sequences.

We now focus on the remaining 6,309 sequences that still display dimerization. To attempt their recovery, we employed the SA framework described in the Dealing with heterodimers: SA section.

This strategy successfully redesigned 4,112 of the 6,309 sequences previously affected by dimerization, completely eliminating all predicted dimers.

**Fig. 7.**
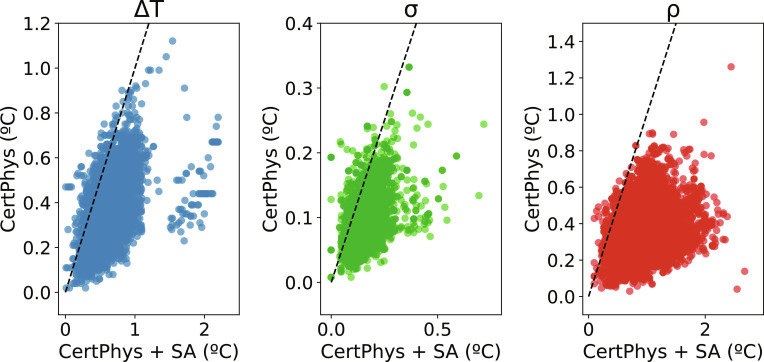
Pairwise comparison of thermal metrics before and after the simulated annealing (SA) repair step for the 4,112 successfully recovered sequences. Lower values indicate higher thermal uniformity. The dashed line represents the y=x diagonal. As expected, most sequences fall below the diagonal, indicating that removing dimers from the design comes at the cost of lowering its thermodynamic quality. Notice that *ρ* is strictly related to the energy function $H^{\prime }$, exactly minimized by CertPhys, so that one could expect that all point should lie below the diagonal. This apparent paradox is caused by the fact that the energy functions Eqs. (1) and (8) are not the same: The latter uses soft constraints for the oligo and overlap lengths, while the exact approach admits no violations. As a consequence, for some sequences, the solution after the SA step not only has no dimers but also presents a better distance to the target temperature. This comes at the price of admitting oligos that slightly violate the constraints on the lengths.

Figure 7 quantifies the impact of the SA layer on the overall thermal quality of the designs, as reported by the metrics $\Delta T_{m}$, *σ*, and *ρ* obtained before and after SA optimization for the 4,112 successfully recovered sequences. The results reveal a modest but consistent increase in all 3 parameters after SA. This confirms that the removal of dimers entails a slight loss in thermal uniformity—an expected trade-off, given that the optimization is constrained to maintain non-interacting oligonucleotides. In some cases, $\Delta T_{m}$ and ρ exceed 2 °C and the designs no longer qualify as Excellent. Nevertheless, in general, the resulting values remain within the range corresponding to good-quality, thermally stable designs. Notice also that the SA solutions can present slight violations of the length constraints imposed by the user, due to the replacement of hard constraints with soft ones in the energy function (see caption of Fig. 7 for more details).

We find that 627 of the 4,112 SA-recovered designs (15.2%) contain at least one oligonucleotide or overlap whose length lies slightly outside the prescribed ranges, for a couple of nucleotides at most; since the soft penalty in Eq. (11) grows with the extent of the violation, these excursions remain small. It is worth mentioning that the resulting designs often present a better thermodynamic quality than those obtained with CertPrime (see Fig. S3), suggesting that the latter were invalid not only due to the presence of dimers but also because of poor values of $\Delta T_{m}$, *σ*, and *ρ*.

Beyond thermal metrics, we also directly assessed the reduction in dimer formation achieved by the SA approach. In particular, we examined both the highest heterodimer melting temperature ($T_{m}^{\max}$) and the total number of dimeric interactions per design before and after SA optimization. These 2 indicators provide complementary perspectives on the effectiveness of the repair process: $T_{m}^{\max}$ captures the severity of the strongest dimer interaction, while the dimer count reflects the overall interaction density across each oligo set.

**Fig. 8.**
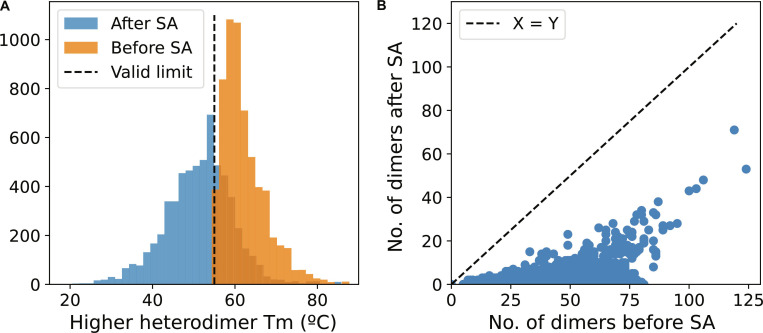
Evaluation of the SA repair step, on the 6,309 dimer-prone sequences. (A) Distribution of the highest heterodimer melting temperature ($T_{m}^{\max}$) in each design before (orange) and after (blue) SA. The dashed line marks the $T^{\mathrm{thr}}=55^{\circ }C$ validity threshold: Those at the left are the 4,112 sequences that become designable after SA. (B) Pairwise comparison of the number of heterodimers per design before and after SA; the dashed line indicates the y=x diagonal. A clear reduction is observed in both metrics after MC optimization, even if the SA layer cannot eliminate dimers completely.

The results, obtained for the set of 6,309 dimer-prone sequences and summarized in Fig. 8, confirm a substantial improvement. Prior to SA, all designs exhibited $T_{m}^{\max}$ values above the 55 °C validity threshold, whereas after SA, the distribution shifted clearly below this limit. Similarly, the number of dimeric interactions per design decreased consistently across the dataset, as shown by the right panel in Fig. 8. These results demonstrate that the SA repair layer not only suppresses high-temperature heterodimers but also significantly reduces their overall frequency, effectively transforming most problematic designs into thermally and structurally viable configurations.

The 2-tiered redesign approach (the exact CertPhys design followed by the SA repair layer) recovered 3,275+4,112=7,387 of the 9,584 dimer-prone sequences, i.e., 77.1% of the dimer-prone subset originally rejected by CertPrime (Fig. 3). Together with the 1,269 sequences rescued on thermal-quality grounds alone, CertPhys (exact + SA, without altering the coding sequence) recovers 8,656 of the 10,853 failed sequences (79.8%).

Despite this success, 2,197 sequences still exhibited at least one dimeric interaction with $T_{m}>T^{\mathrm{thr}}$ after the SA repair step. In these cases, the nucleotide sequence imposed restrictive features that hindered a dimer-free design (most commonly, they are related to long repeats of a single nucleotide, and/or long complementary regions capable of forming stable secondary interactions).

As expected, failures are more likely for longer sequences, where heterodimers can arise from the increased number of (pairs of) oligos in the designs (Fig. S4). However, it can be noticed that at all length, there are sequences that are specially hard to design. So, even if a possible attenuation strategy is to split longer sequences, a safer alternative, if the interest is not in the gene sequence but in the protein it generates, is to apply the codon-changing MC procedure described in the Sequence redesign section, exploring synonymous substitutions that preserve the encoded protein while reshaping the hybridization landscape. We did so for the remaining dimer-prone sequences, whose redesigned DNA sequences were reprocessed with CertPhys under the same experimental and structural conditions as before (also applying the SA repair layer when needed). This final step recovered 1,248 out of 2,197 sequences, which no longer exhibited dimerization above the validity threshold. In the end, only 949 of the initial 10,853 sequences (8.7%) that CertPrime initially failed are left without a good design; these figures could also improve a bit, if we modified the codon-substitution protocol and considered more than one alternative codon sequence per original gene sequence.

Notice that all percentages reported so far are computed with respect to the 10,853 sequences that CertPrime initially failed to design. In the context of the full dataset of 73,424 human coding sequences used to test CertPrime [16], the combination of CertPhys exact and SA stages (without altering the coding sequence) enables the successful design of all but 2,197 targets, i.e., 97.0% of the entire set. Codon redesign yields 1,248 additional recoveries so that a total of 72,475 sequences (98.7%) can be assigned thermally robust, dimer-free oligonucleotide designs.

### Computational performance of CertPhys

We evaluated the computational efficiency of CertPhys using a dataset of sequences ranging from 150 to 1,000 nt in length, as described in the Human genome database preparation section. Each sequence was processed independently, and the wall-clock time required to complete the full oligonucleotide design was recorded.

**Fig. 9.**
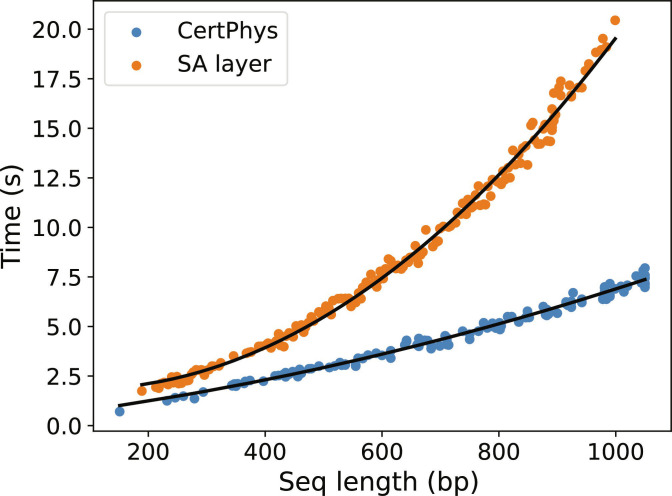
Computational cost analysis of CertPhys and the SA layer. Relationship between sequence length (150 to 1,000 nt) and the computational time required to generate an oligonucleotide design using CertPhys (blue points) and the SA repair layer (orange points). CertPhys timings correspond to the transfer-matrix exact protocol evaluated with the heterodimer-neglecting energy $H^{\prime }$. Solid black lines indicate quadratic regression fits for each method, highlighting the approximate quadratic scaling of computational time with sequence length. All benchmarks were performed on a workstation equipped with a 13th-generation Intel Core i9 processor and 64 GB of RAM, under single-threaded execution and default system conditions.

Figure 9 shows the relationship between sequence length and computation time for the CertPhys design stage. A quadratic regression yields the following empirical model for the execution time *t* (in seconds) as a function of sequence length *L* (in base pairs):

$$t=0.405\;+\;3.59\times 10^{-3}\;L\;+\;2.89\times 10^{-6}\;L^{2}.$$ (16)

The fitted curve reproduces the measured runtimes well. Although the fit includes a quadratic term, its contribution remains small over the range analyzed, and the overall behavior is close to linear for typical sequence lengths. This is consistent with the structure of the algorithm, since the number of oligo lengths evaluated at each position *i* is bounded by the maximum allowed oligo length, and therefore, it does not increase with the total sequence length *L*. In fact, the quadratic contribution only becomes comparable to the linear one for sequences longer than approximately 1.2 × 10^3^.

For the SA repair layer, which is executed independently of CertPhys, a separate quadratic fit was obtained for the computation time (in seconds) versus the length *L* (in base pairs):

$$t=1.99\;\;-\;\;4.6\times 10^{-3}\;\;L\;\;+\;\;2.12\times 10^{-5}\;L^{2}.$$ (17)

In this case, the larger quadratic coefficient reflects the higher computational cost associated with evaluating and iteratively correcting heterodimer interactions across oligo pairs, whose number is expected to grow approximately quadratically with sequence length. Although this repair stage is more computationally demanding than the core CertPhys design step, it is applied only to a limited subset of dimer-prone sequences and represents a necessary trade-off to obtain dimer-free designs. Overall, these benchmarks provide a practical estimate of the runtime required by the different stages of the CertPhys protocol as a function of input size. The computation times of CertPhys are similar to those of CertPrime, while those of the SA layer are significantly larger, yet on the order of some minutes for the range of lengths typically handled in the laboratory.

### Comparison with external tools

Most comparisons in this work are made against CertPrime because the present benchmark is, by construction, the set of sequences that CertPrime failed to design; CertPrime is therefore the most demanding baseline available for this task. CertPrime, in turn, was previously benchmarked head-to-head against widely used external tools—Primerize, PCR OligoMaker, TmPrime, DNAWorks, Gene2Oligo, DeepFirstSearch, and Integrated—and was shown to outperform them in the thermal-quality metrics and in dimer avoidance [16]. A full external head-to-head at the scale of the 10,853-sequence benchmark is not feasible, since several of these tools are no longer available as web servers or as source code, or impose restrictions on input length and experimental conditions. For completeness, we ran CertPhys on the 3 genes (PKB2, GFPuv, and S100A4) used in that external comparison; the results are reported in Table 3, together with the values for the other tools, reproduced from [16]. CertPhys improves even on CertPrime, keeping $\Delta T_{m}\leq 0.22^{\circ }C$ and both *ρ* and *σ* below 0.06 °C for the 3 genes, with a comparable number of oligonucleotides. Since in all 3 cases the local part of CertPhys was sufficient to find a dimer-free solution, we can state that the designs found are the global optimum, given the parameters.

**Table 3.** Comparison of CertPhys with other oligonucleotide-design tools for the PKB2 (length: 1,446 nt), GFPuv (760 nt), and S100A4 (752 nt) genes; *n* is the number of oligonucleotides and $\Delta T_{m}$, *ρ*, and *σ* are the thermal-quality indicators of Eqs. (13) to (15). The CertPhys row was computed in this work; all other rows are reproduced from table 3 of the CertPrime article [16]3. Length-constrained tools (CertPhys, CertPrime, Primerize, PCR OligoMaker) used a maximum oligonucleotide length of 65 nt and a target $T^{*}=60^{\circ }$C; TmPrime, DNAWorks, and Gene2Oligo were taken at $T^{*}=65^{\circ }$C, and DeepFirstSearch and Integrated at $T^{*}=65^{\circ }$C for PKB2 and S100A4 and $55^{\circ }$C for GFPuv. A dash (–) indicates a value not reported in the source; (*) marks a value reported as unavailable.

Tool	PKB2				GFPuv				S100A4			
	*n*	*∆T*_m_	ρ	σ	*n*	*∆T*_m_	ρ	σ	*n*	*∆T*_m_	ρ	σ
CertPhys	38	0.22	0.05	0.05	20	0.10	0.03	0.03	18	0.20	0.04	0.04
CertPrime	40	0.58	0.10	0.10	20	0.73	0.13	0.13	22	0.09	0.09	0.37
Primerize	32	13.64	3.88	3.09	18	3.42	1.29	0.95	18	15.25	4.88	3.74
PCR OligoMaker	30	3.76	2.45	0.97	16	4.43	1.43	1.16	16	5.24	2.15	1.43
TmPrime	60	5.2	2.03	1.2	36	6.8	2.43	1.73	30	8.7	3.04	2.85
DeepFirstSearch	62	9.72	1.45	0.38	–	–	–	–	–	–	–	–
Integrated	62	4.61	0.54	0.49	–	–	–	–	–	–	–	–
DNAWorks	–	–	–	3.7	–	–	–	1.8	–	–	–	3.1
Gene2Oligo	–	–	–	7.6	–	–	–	–	–	–	–	${}^{*}$

### Experimental results

To complement the computational evaluation and assess the practical reliability of CertPhys designs, we conducted a series of experimental validations. Specifically, we aimed to determine whether the algorithm’s predictions—particularly regarding dimer formation—correlate with experimental outcomes. To this end, we selected a set of 14 sequences of varying lengths, ranging from short templates (150 nt) to long constructs (up to 865 nt). All sequences were processed using CertPhys to generate oligonucleotide sets predicted to be free of dimer interactions.

Before proceeding with experimental validation, for each sequence, we evaluated some global property (length and GC content), local compositional extremes (the partial GC content in the most biased 50-nt windows), as well as the parameters describing the resulting oligonucleotide sets generated by CertPhys. These include oligo and overlap lengths, their respective GC content ranges, and the thermal quality indicators $\Delta T_{m}$, *σ*, and *ρ*, defined in section 2.6.2.

Table 4 reveals that all 14 designs met the strictest thermal classification thresholds and qualified as “Excellent”, with values of $\Delta T_{m}$ below 0.5 °C and extremely low temperature variability and deviation scores. Additionally, potential dimer formation was evaluated by computing the melting temperature $T_{m}^{\mathrm{Dim}}$ of the strongest self-interacting oligo or most stable heterodimeric pair. In all cases, $T_{m}^{\mathrm{Dim}}$ values remained well below typical annealing temperatures, suggesting low risk of undesired interactions under standard assembly conditions.

**Table 4.** Characteristics of the sequences used for computational and experimental tests, and parameters of the corresponding oligonucleotide designs produced by CertPhys. *L* is the sequence length, *n* is the number of oligonucleotides in the design, %CG is the CG content, and %CG_50_ represents the GC content of the 50-nucleotide region in the sequence, with the highest and lowest GC content. The column L^(O)^ reports the maximum and minimum lengths of the oligos, while L^(ov)^ reports the maximum and minimum lengths of the overlap regions. %CG${}^{\left(O\right)}$ reports the maximum and minimum CG percentages of the oligos, and %CG^(ov)^ reports the maximum and minimum CG percentages of the overlap regions. The indicators $\Delta T_{m}$, *ρ*, and *σ* were introduced in Eqs. (13), (14), and (15), respectively. $T_{m}^{\mathrm{Dim}}$ is the highest temperature between the melting temperature of the oligonucleotide with the strongest self-interaction, and the melting temperature of the pair of oligonucleotides that most strongly interact with each other (forming the most stable heterodimer).

Ident	*L*	*n*	%*CG*	%*CG*_50_	*L* ^(O)^	*L* ^(ov)^	%*CG*^(O)^	%*CG*^(ov)^	∆*T*_m_	σ	*ρ*	$T_{m}^{\mathrm{Dim}}$
Seq_1	150	4	46.0	36.0–56.0	51–58	22–27	41.4–50.9	37.0–50.0	0.08	0.04	0.03	33.36
Seq_2	180	4	47.8	34.0–60.0	55–65	19–22	39.3–55.4	50.0–57.9	0.07	0.04	0.22	37.07
Seq_3	180	4	48.3	38.0–56.0	40–55	19–23	37.0–54.4	43.5–57.9	0.49	0.19	0.16	37.10
Seq_4	180	4	46.1	40.0–56.0	43–55	19–25	39.5–54.6	41.7–63.2	0.42	0.15	0.13	24.64
Seq_5	180	4	57.8	50.0–68.0	38–52	17–22	46.2–65.8	52.4–70.6	0.08	0.03	0.07	35.41
Seq_6	184	4	50.5	42.0–66.0	44–52	18–22	45.7–60.9	45.5–63.2	0.21	0.08	0.07	33.24
Seq_7	190	6	42.1	34.0–52.0	48–53	20–26	34.6–50.0	38.5–55.0	0.38	0.18	0.14	23.33
Seq_8	210	6	42.4	28.0–54.0	51–62	20–31	34.6–50.9	32.3–55.0	0.12	0.06	0.05	31.32
Seq_9	284	8	57.8	46.0–68.0	42–62	15–20	50.0–66.1	52.6–80.0	0.20	0.08	0.06	47.20
Seq_10	593	14	53.8	40.0–70.0	50–63	14–30	43.1–76.5	33.3–80.0	0.18	0.06	0.05	47.09
Seq_11	631	16	47.9	28.0–72.0	43–64	16–31	35.0–63.5	29.0–75.0	0.48	0.10	0.06	44.25
Seq_12	675	16	55.1	38.0–74.0	42–61	15–25	43.4–69.4	40.0–80.0	0.27	0.09	0.08	50.70
Seq_13	714	18	50.0	30.0–68.0	49–64	15–34	34.4–76.5	26.5–75.0	0.41	0.10	0.08	51.20
Seq_14	865	22	51.1	34.0–64.0	43–60	15–32	35.2–60.3	31.3–75.0	0.30	0.08	0.07	43.61

This comprehensive characterization suggested that CertPhys generated high-quality oligonucleotide designs for all selected sequences, paving the way for their synthesis and in vitro validation.

**Fig. 10.**
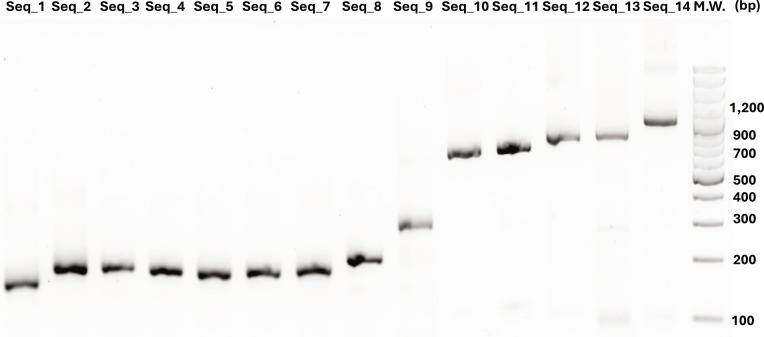
Agarose gel electrophoresis profiles for the 14 oligonucleotide designs produced by CertPhys. Each lane corresponds to one of the experimentally tested sequences (Seq 1 to Seq 14). All samples show a single, well-defined band of the expected size, with no evidence of secondary bands or smearing. This indicates the absence of undesired dimer formation—either homo- or heterodimers—thus experimentally confirming the accuracy of CertPhys dimer predictions.

The 14 assemblies were synthesized and analyzed by agarose gel electrophoresis. The resulting profiles, shown in Fig. 10 and Fig. S5, display a single, sharp band for each sequence, with no detectable secondary bands, smearing, or laddering. This is consistent with the CertPhys designs being free from disruptive homo- and heterodimers under these experimental conditions; in addition, Sanger sequencing confirmed the intended assembled sequence in all cases (sequences not shown, for confidentiality reasons).

The agreement between computational predictions and gel-based validation supports the use of CertPhys for producing clean, thermodynamically consistent oligonucleotide designs across the tested range of sequence lengths and compositional biases.

On the other hand, we stress that the large-scale evidence for the robustness of the method stands upon the in silico evaluation over the 73,424-sequence dataset; the wet-lab assays, which are necessarily limited to a small sample, represent a proof-of-concept of how smoothly the theoretical predictions translate to experimental evidence.

Indeed, several limitations of this validation should be acknowledged. First, it is worth mentioning that the melting temperatures are computed with Primer3 under the ionic conditions of Table 1 and do not explicitly account for 3% DMSO of the PCR mixture, nor for kinetic effects, secondary structures, polymerase-specific behavior, or synthesis-chemistry artifacts that may also affect assembly. Therefore, even very good values of the thermal metrics do not necessarily imply accordingly good experimental results, and this is why a positive outcome, even for a limited sample of 14 sequences, is relevant to establish a connection between in silico and real results.

On the other hand, we did not include dimer-positive negative controls, since the aim was not to systematically characterize sequences predicted to fail, but to confirm that constructs designed to avoid dimerization behave as intended, in the framework of a reliable experimental assay, whose ability to reveal dimer bands had already been demonstrated for failing designs in CertPrime [16]. Indeed, agarose gel electrophoresis resolves gross homo- and heterodimers and mis-assembled products by size; even if low-level dimer formation may fall below its detection threshold, the single bands of the expected size, together with the Sanger verification of the assembled sequences, argue against any substantial off-target product.

### CertPhys web server

The CertPhys tool is accessible at https://oligodesign.bifi.es upon registration. CertPhys is implemented in Python, using Streamlit as the web framework, and runs on a standard Ubuntu environment. Melting temperatures are computed with the primer3-py package (version 2.0.3).

The web interface enables users to input the target sequence and specify experimental conditions; a simple manual is provided on the left of the web page. The design process is then executed starting by finding the optimal solution (neglecting heterodimers) and implementing the SA layer in case such solution presents heterodimers and needs to be discarded. Once the computation is completed, the resulting design is evaluated and its features are summarized in a table, ready for download. We have opted for the simplest solutions both for the interface and for data management: For privacy and security reasons, uploaded sequences are not stored on our servers; they are automatically deleted once downloaded by the user, ensuring full compliance with legal requirements.

## Conclusions

In this work, we presented CertPhys, a novel method for the design of overlapping oligonucleotides based on a statistical physics approach. The protocol starts by mapping any possible oligo-design on a configuration of an ad hoc statistical physics system and by finding the exact, low-temperature solution, using an energy function that encodes all the relevant features that a good design has to satisfy. Such solution corresponds to the globally optimal design, when spurious interactions between distant oligos, originating heterodimers, can be neglected. If the resulting design presents heterodimer, an SA protocol is applied to find an alternative dimer-free solution. For the sequences that are still affected by heterodimers, we propose a codon-redesign approach, for the cases in which such change in the original gene sequence makes sense.

We stress that the novelty of the method is associated to several features that distinguish it from the common heuristic approaches: Recasting the problem in statistical physics language allows to clearly separate local terms in the cost function [$H^{\prime }$ in Eq. (1)] from the nonlocal term *V* penalizing hetero-dimers, and, in a sense, regarding the second as a perturbation of the first term. Second, it prompts for the usage of statistical physics tools to find the exact global solution of the former term, as low-temperature averages of some observable. Even if the minimum of the energy function $H^{\prime }$ could be found by more standard approaches (e.g., dynamic programming), the “temperature” parameter in the physical approach naturally prompts for the exploration of suboptimal configurations, which can be used as “educated” initial condition for the simulated-annealing repair of the genuinely nonlocal heterodimer interactions—a problem conjectured to be computationally hard [21]—for which we make no claim of global optimality.

CertPhys overcomes many of the limitations associated with heuristic-based methods such as CertPrime. We demonstrated that CertPhys is capable of finding a global, dimer-free optimal design for 41.9% of the sequences that CertPrime failed to design—either due to dimer formation or thermal inconsistencies. This figure raises to 79.8% after the SA step to remove heterodimers, reaching 91.3% if codon redesign is allowed for the most challenging sequences. Notice that the latter figures could potentially be higher (we did not invest significant effort into optimizing the SA and codon redesign approaches); however, it is also likely that there might be gene sequences that are impossible to design while respecting all constraints. Such cases should be treated on a case-by-case basis, deciding which features to keep and specially tailoring the parameters and algorithms for them—something that is out of the scope of the present work.

In all our tests, CertPhys consistently achieved low values for key thermal metrics ($\Delta T_{m}$, σ, and ρ) and generated oligonucleotide designs with minimal risk of undesired interactions.

Experimental validation was consistent with these predictions: all 14 test sequences, spanning a wide range of lengths and GC contents, yielded clean, single-band assemblies on agarose gels with no detectable dimer formation, and their assembled sequences were confirmed by Sanger sequencing. These results indicate that CertPhys translates effectively into reliable experimental outcomes, within the limitations noted above.

The computational cost of CertPhys designs is similar to that associated with our heuristic method, CertPrime; thus, its efficiency does not come at the cost of a heavy computational burden. While the SA repair layer is more demanding, it is only required for challenging sequences.

Altogether, the ability of CertPhys to produce robust and synthesis-ready designs—especially in challenging sequence contexts—makes it a valuable tool for gene synthesis applications in both research and industry.

## Data Availability

The CertPhys web server is publicly available at https://oligodesign.bifi.es. It provides access to the CertPhys pipeline as well as to the SA and codon redesign layers described in this work. The website includes an interactive interface to run analyses and inspect outputs, together with a user manual detailing the required inputs, parameter settings, and interpretation of the results. The benchmark sequence identifiers and the corresponding CertPhys and CertPrime design outputs and runtime tables are available from the authors on reasonable request.
