# Supplementary material for: A Physics-Inspired Approach to Improve Oligonucleotide Design and Gene Synthesis
Source: Comput Struct Biotechnol J. 2026 Aug 6;35(1):0189. doi: 10.34133/csbj.0189 (PMC13443015; doi:10.34133/csbj.0189)
Supplement: Supplementary file 1 [file csbj.0189.f1.pdf]

A physics-inspired approach to improve oligonucleotide design and gene synthesis: Supplementary Information

David Luna-Cerralbo[a,b,c], Ana Serrano[c], Fadi Hamdan[c], Juan Martínez-Oliván[c], Pierpaolo Bruscolini[a,b,*]

[a] *Departamento de Física Teórica, Facultad de Ciencias, Universidad de Zaragoza, c/ Pedro Cerbuna s/n, Zaragoza, 50009, Spain*
[b] *Instituto de Biocomputación y Física de Sistemas Complejos (BIFI), Universidad de Zaragoza, c/ Mariano Esquillor s/n, Zaragoza, 50018, Spain*
[c] *Certest Pharma, Certest Biotec S.L, Polígono Industrial Río Gallego II, Calle J, 1, San Mateo de Gállego, 50840, Spain*

S1. Supplementary Methods

S1.1. Model constraints

As seen in the main text, not all possible combinations of $\xi_i = (x_i, v_i, \sigma_i)$ at any site i correspond to meaningful designs, and a series of consistency constraints must be introduced, in order to establish a one to one relation between possible designs and allowed configurations:

1. Start–End conditions:.

- at $i = 1$, $x_1 = 0$, $v_1 = 0$, $\sigma_1 = 0$;
- at $i = L$, $x_L \in [0, l^M - 1]$, $v_L = 0$, $\sigma_L = 1$

Notice that the starting strand is arbitrary, but leaving the freedom to start at both strands would be redundant, so we force the first oligo of the design to stay in the sense strand. For biotechnological efficiency, we ask the last oligo to stay in the opposite strand.

*Corresponding authors

Email addresses: dluna@unizar.es (David Luna-Cerralbo), anaserra1979@gmail.com (Ana Serrano), fhamdan@certest.es (Fadi Hamdan), jmartinez@certest.es (Juan Martínez-Oliván), pier@unizar.es (Pierpaolo Bruscolini)

2. On-site constraints:.

- $v_i \leq \min(x_i, O^M)$, the "accumulated overlap" at i cannot exceed the minimum between the "accumulated length" and the maximum allowed overlap length;

- $x_i \leq \min\big(i-1,\ l^M - 1\big)$ the accumulated length is limited by the maximal length allowed for the oligos.

- State $(x_i,\ v_i,\ \sigma) = \big(l^M - 1,\ 0,\ \sigma\big)$ is forbidden for $i \leq L-1$ and is only admitted if $i = L$, since it implies no overlap with the following oligo, which is only allowed at the end.

3. Constraints on neighbouring sites:. Let's consider the pair of sites $(i-1, i)$; it is convenient to deal separately the case of the interior sites $i < L$ from that of the terminal site $i = L$. For the former, three possible cases are possible:

- an oligo of the design ends at $i-1$:

$$(x_i, v_i, \sigma_i) = (v_{i-1}, \delta, 1 - \sigma_{i-1}), \tag{S1}$$

  with $\delta = 0, 1$ corresponding to the the case of gapped or ungapped design, $O^m \leq v_{i-1} \leq O^M$, and $x_{i-1} \geq l^m - 1$, if we want to enforce a minimal oligo length l^m, and a minimal (O^m) and maximal overlap length (O^M);

- the oligo continues at i, and i is outside the overlap region with the following oligo (i.e., this is only possible if we allow gapped designs):

$$(x_i, v_i, \sigma_i) = (x_{i-1} + 1,\ 0,\ \sigma_{i-1}) \tag{S2}$$

  with $v_{i-1} = 0$;

- the oligo continues at i, and i is inside the overlap (hybridization) region with the following oligo:

$$(x_i, v_i, \sigma_i) = (x_{i-1} + 1,\ v_{i-1} + 1,\ \sigma_{i-1}) \tag{S3}$$

  with $0 \leq v_{i-1} \leq O^M - 1$.

The terminal $i = L$ case is similar to the previous one, but now only the first two cases are possible (and, of course, the last oligo ends at L):

- an oligo of the design ends at $L-1$:

$$(x_L, v_L, \sigma_L) = (v_{L-1}, 0, 1-\sigma_{L-1}), \tag{S4}$$

  with $O^m \leq v_{L-1} \leq O^M$ $x_{L-1} \geq l^m - 1$, and $x_L \geq l^m - 1$;

- the oligo continues and ends at L:

$$(x_L, v_L, \sigma_L) = (x_{L-1}+1,\ 0,\ \sigma_{L-1}) \tag{S5}$$

  with $v_{L-1} = 0$ and $l^m - 1 \leq x_L \leq l^M - 1$.

S1.2. Simulated Annealing.

S1.2.1. Elementary move set.

The Monte Carlo simulation at each temperature is built on the following set of local (number-preserving) and non-local (number-changing) moves:

1. Overlap-boundary moves. These local perturbations shift the boundaries' positions by $\Delta \in \{\pm 1, \pm 2, \pm 3\}$ nucleotides, provided that their order is preserved:

- *Upstream window slide:* $b_{i,1} \leftarrow b_{i,1}+\Delta,\ b_{i,2} \leftarrow b_{i,2}+\Delta$ (preserves $|O_i^{\uparrow}| = b_{i,2} - b_{i,1}$).

- *Upstream length tweak:* $b_{i,1} \leftarrow b_{i,1}+\Delta$ or $b_{i,2} \leftarrow b_{i,2}+\Delta$ (changes $|O_i^{\uparrow}|$ by Δ).

- *Core slide:* $b_{i,2} \leftarrow b_{i,2}+\Delta,\ b_{i,3} \leftarrow b_{i,3}+\Delta$ (preserves the core length).

- *Downstream length tweak:* $b_{i,3} \leftarrow b_{i,3}+\Delta$ or $b_{i,4} \leftarrow b_{i,4}+\Delta$ (preserves $|O_i^{\downarrow}| = b_{i,4} - b_{i,3}$).

- *Downstream window slide:* $b_{i,3} \leftarrow b_{i,3}+\Delta,\ b_{i,4} \leftarrow b_{i,4}+\Delta$ (changes $|O_i^{\downarrow}|$ by Δ).

2. Oligo addition/removal. Moves that modify the total number of oligos by ± 2, carried out according to the scheme shown in Figure 2 in the main text, that we reproduce here for the sake of clarity as Fig. S1:

Remembering that *Join* removes an intermediate core and its flanking overlaps, while *Split* inserts a new core and two overlaps within the core of an existing oligo, the main challenge here is to determine the values that will serve as the new boundary positions, $b_{i,1}$, $b_{i,2}$, $b_{i,3}$, and $b_{i,4}$, and, crucially,

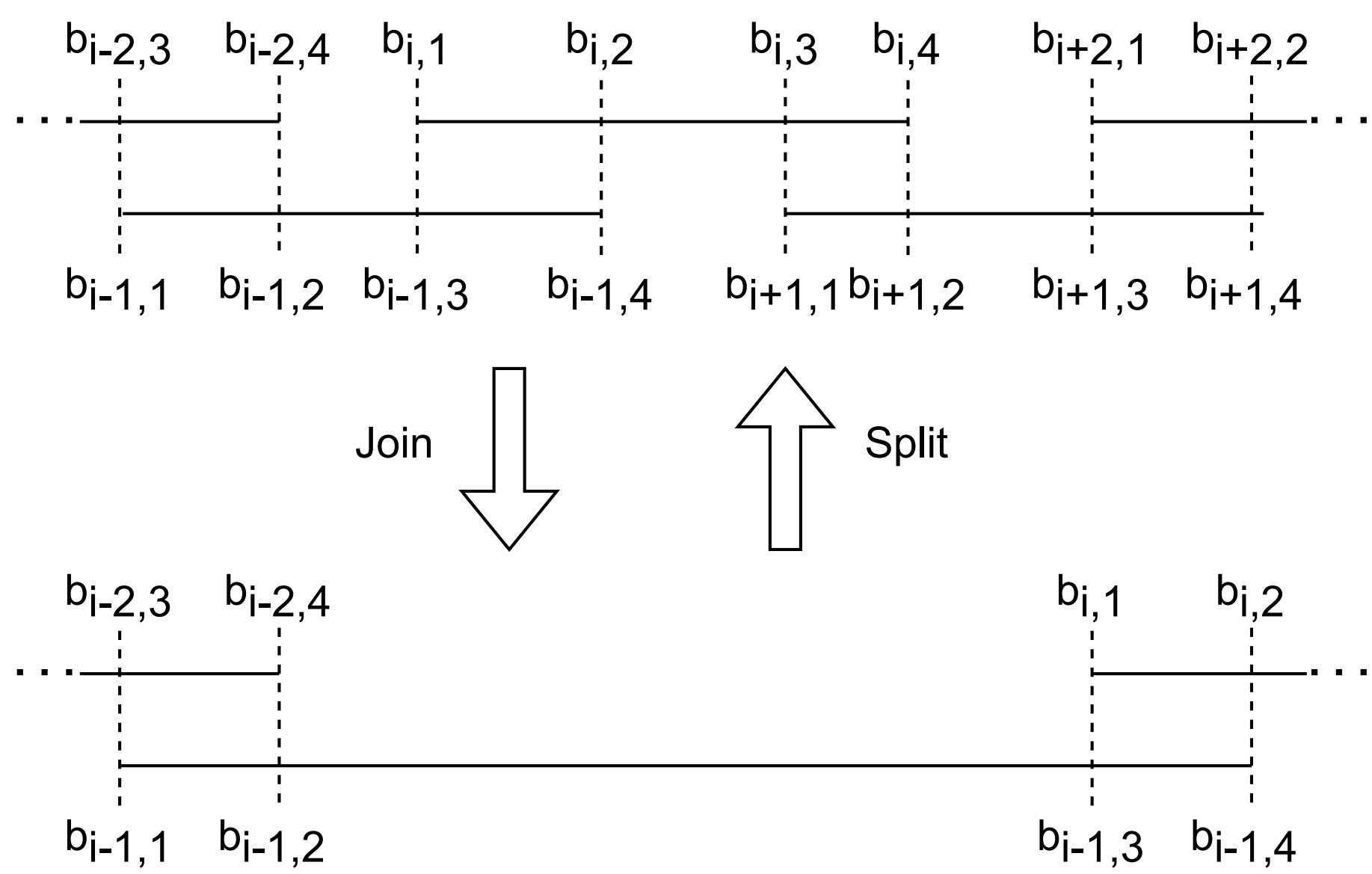

Figure S1: Schematic representation of the relative positions involved in the merge and split oligo movements.

to do so efficiently. Our strategy begins by fixing $b_{i,4}$. To achieve this, we identify the position at which an oligo is most likely to terminate:

$$b_{i,4} = \arg\max \left(\sum_{h=b_{i-2,4}+1}^{j-1} p(o^{\sigma}_{hj}) \right) \quad \forall j \in [b'_{i-2,4} + 1 < j < b'_{i,1}]$$

where $p(O^{\sigma}_{hj})$ denotes the probability that an oligo starts at position h and ends at position j on strand σ, computed using Eq. 7. Once the new value $b_{i,4}$ has been fixed, we determine $b_{i,1}$ by:

$$b_{i,1} = \arg\max \left(p(o^{\sigma}_{j,b_{i,4}})) \right) \quad \forall j \in [b'_{i-2,4} < j < b_{i,4} - 1]$$

With $b_{i,1}$ fixed, the next step is to determine $b_{i,2}$. To do so, we continue to apply the same strategy:

$$b_{i,2} = \arg\max \left(p(o^{\sigma}_{b_{i-1,1},j}) \right) \quad \forall j \in [b_{i,1} < j < b_{i,4} - 1]$$

and finally, we fix $b_{i,3}$ as:

$$b_{i,3} = \arg\max\left(p(o^{\sigma}_{j,b_{i-1,4}})\right) \quad \forall j \in [b_{i,2} < j < b_{i,4}]$$

In this way, we obtain the positions $b_{i,1}$, $b_{i,2}$, $b_{i,3}$, and $b_{i,4}$ that define the split movement.

To select which move to perform, we first choose the *move class*: either a *local* move or a *split/join* move. The probability of selecting each class is proportional to the number of moves it contains—five for local moves and two for split/join moves. Hence, the probability of performing any given local move is

$$p^{(\mathrm{loc})} = \frac{5}{7 \cdot 5}.$$

However, each local move also requires selecting a value of Δ. Therefore, letting n denote the number of oligos, the probability of performing a specific local move on oligo i with a particular value of Δ is

$$p^{(\mathrm{loc},\,\Delta)} = \frac{1}{n \cdot 7 \cdot 6}.$$

By contrast, the probability of selecting a split/join move is

$$p^{(S)} = p^{(J)} = \frac{2}{7 \cdot 2}.$$

We refer to $p^{(\mathrm{loc},\,\Delta)}$, $p^{(J)}$, and $p^{(S)}$ as the *background probabilities* of the respective moves.

The combination of local slides/tweaks and split/merge moves makes the Markov chain irreducible and aperiodic over the feasible state space, thereby allowing exploration of any admissible segmentation.

Note that the moves above preserve the parity of the total number of oligos n. Therefore, once an initial configuration with even n is chosen, this property is maintained at every step.

S1.2.2. Monte Carlo acceptance rule.

Move proposal $\boldsymbol{\eta} \to \boldsymbol{\eta}'$ drawn from kernel q are accepted according to a Metropolis-Hastings criterion:

$$\alpha(\boldsymbol{\eta} \to \boldsymbol{\eta}') = \min\left\{1, \frac{q(\boldsymbol{\eta}' \to \boldsymbol{\eta})}{q(\boldsymbol{\eta} \to \boldsymbol{\eta}')} e^{-\frac{1}{\tau}(H_{MC}(\boldsymbol{\eta}') - H_{MC}(\boldsymbol{\eta}))}\right\} \tag{S6}$$

In this framework, we can take advantage of the knowledge of the exact solution for the simplified model, using Eq. 7 as a background probability associated to the elementary moves proposal. Thus, we write

$$q(\boldsymbol{\eta} \to \boldsymbol{\eta}') = p^{\text{bkgr}} w(\boldsymbol{\eta} \to \boldsymbol{\eta}') \tag{S7}$$

where p^{bkgr} is the background probability associated to the elementary move connecting the two states, and $w(\boldsymbol{\eta} \to \boldsymbol{\eta}')$ is the product of terms of the kind $p(o^{\sigma}_{b_{i,1},b_{i,4}})$ for each oligo i on strand σ that appears in state $\boldsymbol{\eta}'$ and was not present in $\boldsymbol{\eta}$, and $(1 - p(o^{\sigma}_{b_{i,1},b_{i,4}}))$ for each oligo i on strand σ that is contained in state $\boldsymbol{\eta}$ and disappears in $\boldsymbol{\eta}'$.

S1.2.3. Simulated Annealing protocol

The SA procedure consists of 20 Metropolis–Hastings Monte Carlo simulations performed at decreasing temperatures τ_k, from $\tau_0 = 100$ to $\tau_{19} = 0.01$, using geometric cooling

$$\tau_k = 100 \left(\frac{0.01}{100} \right)^{k/19}, \qquad k = 0, \ldots, 19,$$

and $n_S = 20{,}000$ MC steps at each temperature. For each sequence a single annealing run is performed, initialised from the optimal design obtained with the exact transfer-matrix solution of H'. Because that design is already optimal for the local model and only the (typically few) heterodimer-prone overlaps need to be repaired, the search is essentially local and its outcome is robust to the precise cooling schedule. Cooling proceeds down to $\tau_{19} = 0.01$, where the acceptance of energy-increasing moves is negligible and the configuration is effectively frozen.

Each step starts by selecting uniformly at random one oligo from the current configuration, followed by a uniform random choice of the move class (local vs. non-local), and then a uniform random choice of a move within the selected class. Finally, for local moves, a value of Δ is chosen uniformly at random. If the proposed move violates any constraint (e.g., boundary crossing), it is discarded and a new proposal is generated. The energy change associated with the selected move is then computed, and the move is accepted or rejected according to Eq. S6.

S2. Supplementary Figures

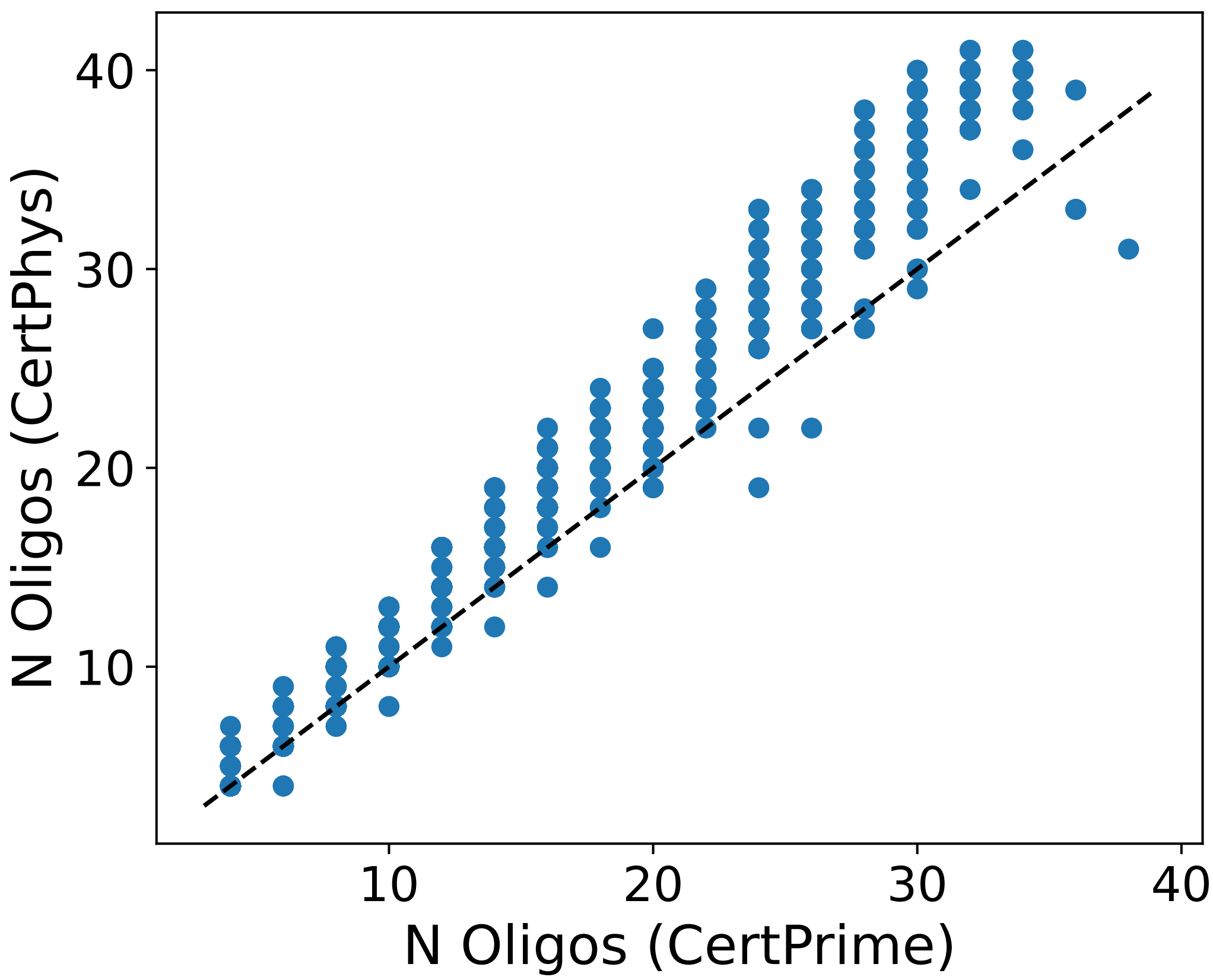

Figure S2: Differences in the number of oligos in the best designs by CertPhys and CertPrime, for the subset of 1,269 sequences for which CertPrime could not provide a satisfactory solution. CertPhys consistently uses a greater number of shorter oligos, as reflected by the upward shift from the identity line (dashed).

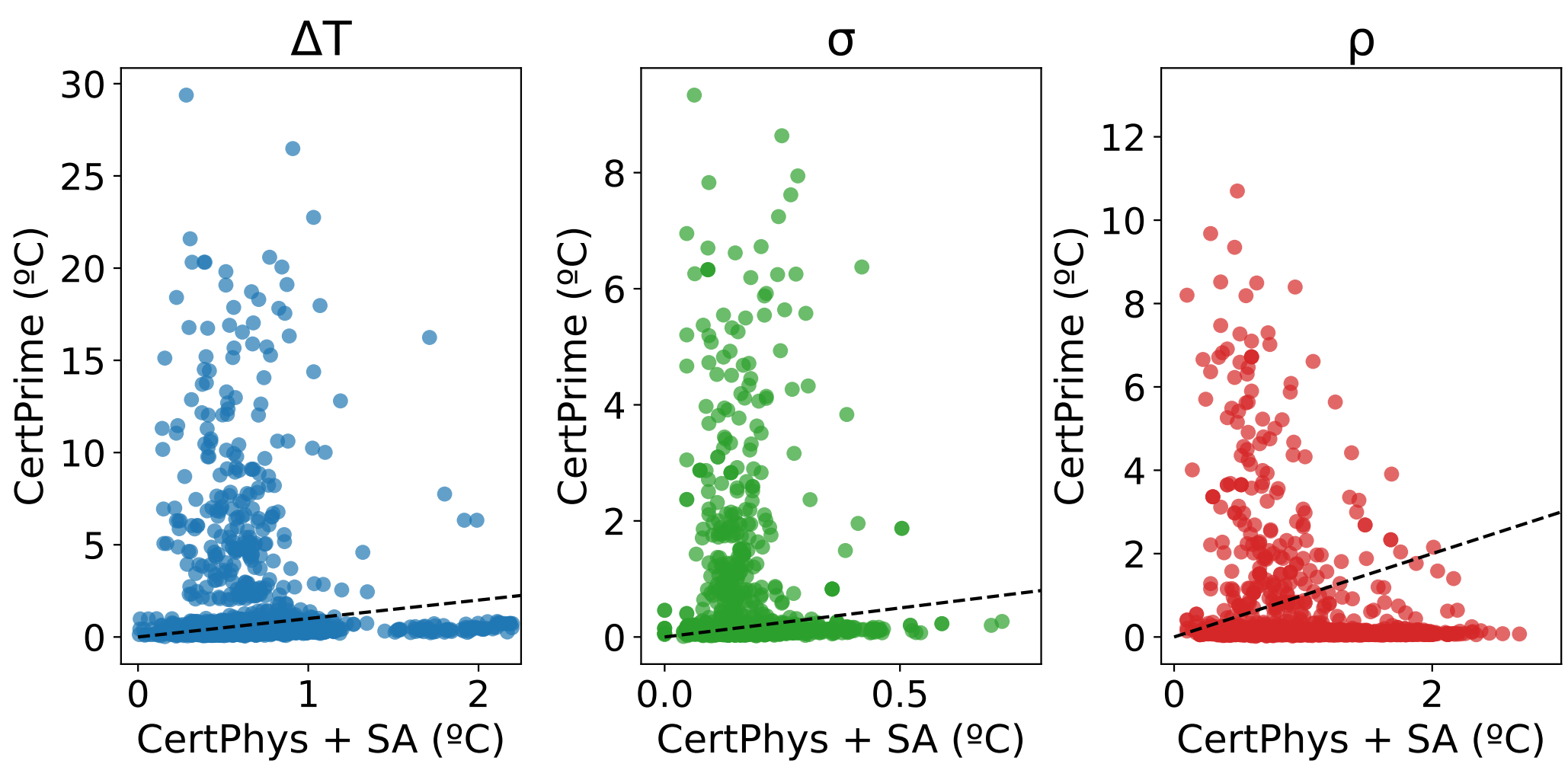

Figure S3: Pairwise comparison of thermal quality metrics between CertPrime (y-axis) and CertPhys+SA (x-axis) for the 4,112 sequences recovered through the Simulated Annealing Monte Carlo repair layer. Lower values indicate higher thermal uniformity and thus improved design quality.

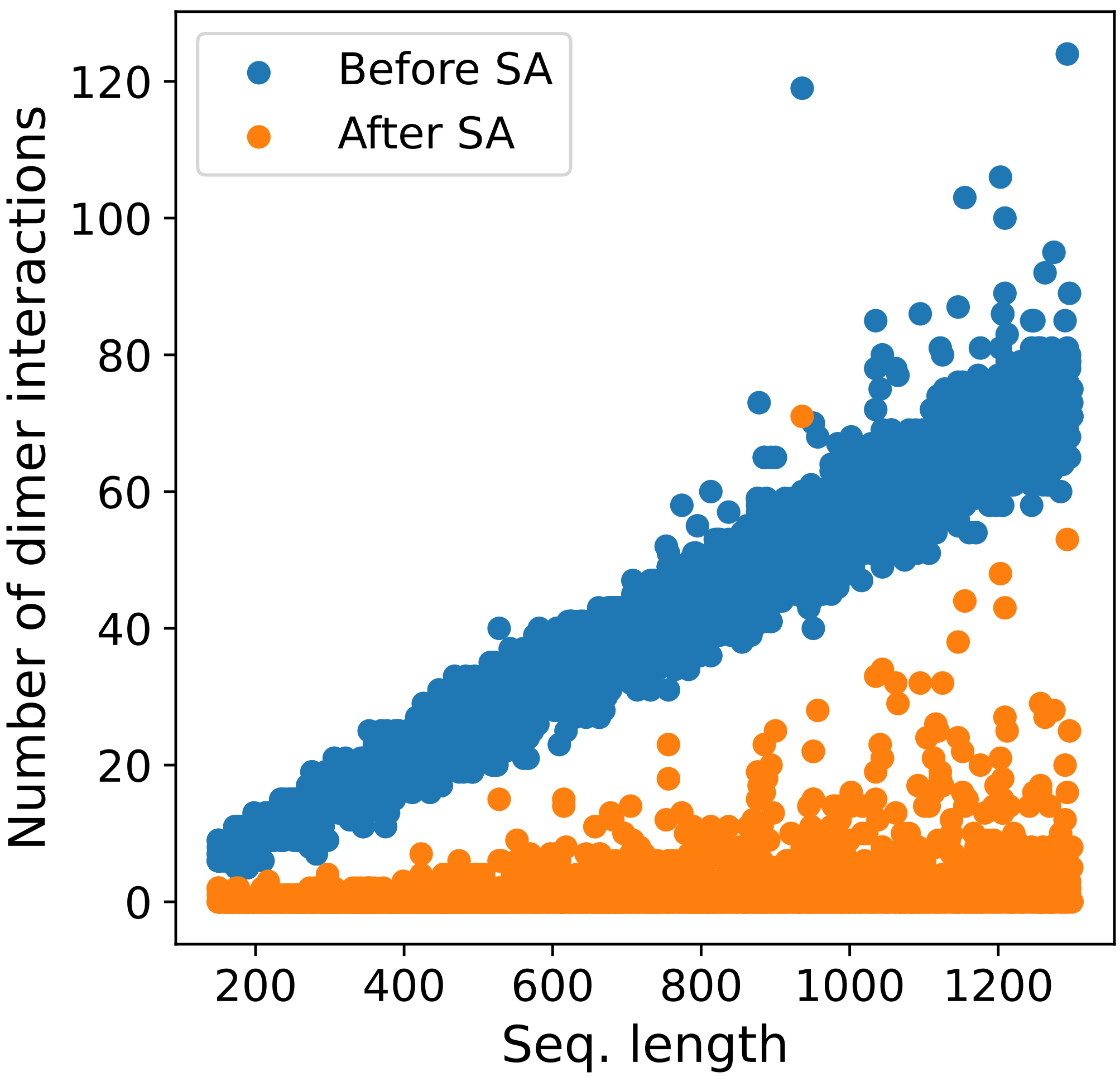

Figure S4: Number of dimer interactions (with melting temperature higher than T^{thr}) vs sequence length L: it appears that the number of dimers increase roughly linearly with sequence length. After the SA step to remove dimers, we still see that longer sequence are more dimer-prone, even if the number of dimers has greatly decreased. We also see that, at all lengths, there are especially hard sequences that are outliers, and present several dimers that could not be removed

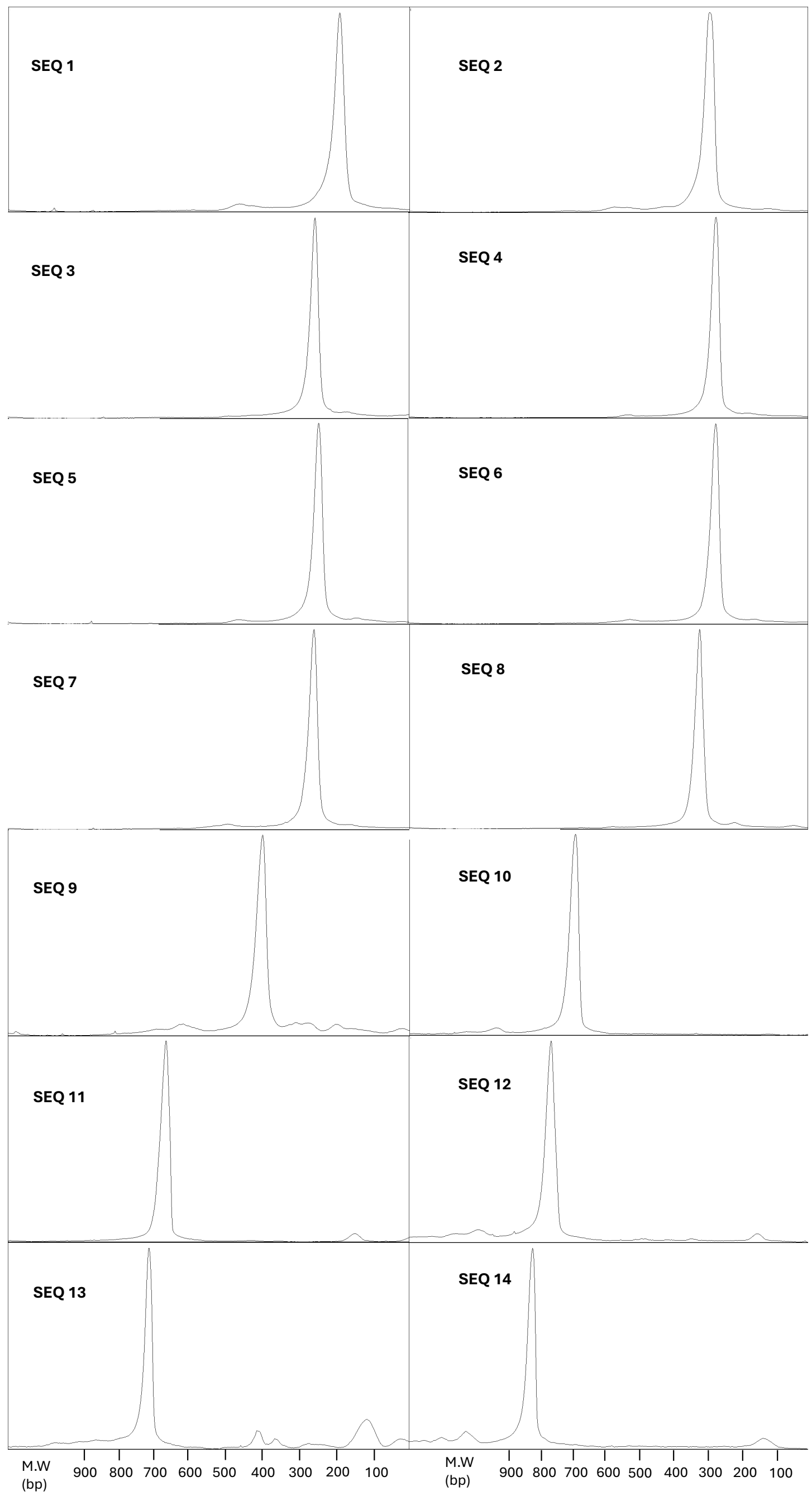

Figure S5: Densiometric intensity profiles of the 14 oligonucleotide assemblies designed by CertPhys, derived from the corresponding electrophoresis profiles in Figure 10 of the main text; Each profile corresponds to a single DNA construct assembled from overlapping oligonucleotides. The height of the peak at the respective molecular weight correlates with the intensity of each band in that figure. An optimal amplification is represented by a sharp, narrow peak aligned with the expected molecular weight of each gene sequence. All lanes show a single, well-defined peak at the expected molecular weight, with no detectable secondary bands, smearing, or laddering. These results confirm the absence of undesired dimer formation —either homo- or heterodimers— and experimentally validate the accuracy of CertPhys's computational predictions. Molecular weight markers (MW) are included for reference at the bottom.
